# In-Situ Synthesis and Characterization of Nanocomposites in the Si-Ti-N and Si-Ti-C Systems

**DOI:** 10.3390/molecules25225236

**Published:** 2020-11-10

**Authors:** Maxime Balestrat, Abhijeet Lale, André Vinícius Andrade Bezerra, Vanessa Proust, Eranezhuth Wasan Awin, Ricardo Antonio Francisco Machado, Pierre Carles, Ravi Kumar, Christel Gervais, Samuel Bernard

**Affiliations:** 1 CNRS, IRCER, UMR 7315, University of Limoges, F-87000 Limoges, France; maxime.balestrat@unilim.fr (M.B.); abhijeet.lale@unilim.fr (A.L.); andrevbezerra@gmail.com (A.V.A.B.); pierre.carles@unilim.fr (P.C.); 2 Institut Europeen des Membranes (IEM), UMR 5635 (CNRS-ENSCM-UM2), Universite Montpellier 2, Place E. Bataillon, F-34095 Montpellier, France; vanessa.proust6@gmail.com; 3 Chemical Engineering, Federal University of Santa Catarina, Florianópolis 88010-970, Brazil; ricardo.machado@ufsc.br; 4 Laboratory for High Performance Ceramics, Department of Metallurgical and Materials Engineering, Indian Institute of Technology-Madras (IIT Madras), Chennai 600036, India; eranezhuth@gmail.com (E.W.A.); nvrk@iitm.ac.in (R.K.); 5 Laboratoire Chimie de la Matière Condensée de Paris, Collège de France, CNRS, Sorbonne Université, 4 Place de Jussieu, 75005 Paris, France; christel.gervais_stary@sorbonne-universite.fr

**Keywords:** polymer-derived ceramics, nanocomposites, TiN, TiC, Si_3_N_4_, SiC, structural properties

## Abstract

The pyrolysis (1000 °C) of a liquid poly(vinylmethyl-*co*-methyl)silazane modified by tetrakis(dimethylamido)titanium in flowing ammonia, nitrogen and argon followed by the annealing (1000–1800 °C) of as-pyrolyzed ceramic powders have been investigated in detail. We first provide a comprehensive mechanistic study of the polymer-to-ceramic conversion based on TG experiments coupled with in-situ mass spectrometry and ex-situ solid-state NMR and FTIR spectroscopies of both the chemically modified polymer and the pyrolysis intermediates. The pyrolysis leads to X-ray amorphous materials with chemical bonding and ceramic yields controlled by the nature of the atmosphere. Then, the structural evolution of the amorphous network of ammonia-, nitrogen- and argon-treated ceramics has been studied above 1000 °C under nitrogen and argon by X-ray diffraction and electron microscopy. HRTEM images coupled with XRD confirm the formation of nanocomposites after annealing at 1400 °C. Their unique nanostructural feature appears to be the result of both the molecular origin of the materials and the nature of the atmosphere used during pyrolysis. Samples are composed of an amorphous Si-based ceramic matrix in which TiN_x_C_y_ nanocrystals (x + y = 1) are homogeneously formed “in situ” in the matrix during the process and evolve toward fully crystallized compounds as TiN/Si_3_N_4_, TiN_x_C_y_ (x + y = 1)/SiC and TiC/SiC nanocomposites after annealing to 1800 °C as a function of the atmosphere.

## 1. Introduction

Silicon carbide (SiC) and silicon nitride (Si_3_N_4_) are technologically relevant high-performance ceramics which attract nowadays strong interest for driving the development of energy, environment and health sectors [1,2,3,4]. By the synergistic cooperation between SiC or Si_3_N_4_—as a matrix—and a dispersed ceramic nanophase of different chemical composition—as a nano-precipitate—nanocomposites with peculiar physical and chemical properties (e.g., mechanical, electrical, optical, catalytic, etc.) that are of high scientific and technological importance can be generated [5,6,7,8].

As developed functional properties closely depend on the synthesis route of these materials. For this purpose; ceramic processing methods based on molecular engineering and precursor chemistry are well appropriate approaches to design nanocomposites that can reach performances far beyond those developed by more conventional synthesis routes. In general, such nanocomposites exhibit improved properties when compared to those prepared via classical high temperature metallurgical techniques because of the homogeneous distribution of the nanophase within the matrix (i.e., absence of agglomeration of the nano-precipitates), the small size of the nano-precipitates (no sintering because of the generally low temperature of preparation) and the lack of undesirable elements. A very convenient precursor route to produce these materials in non-oxide ceramic systems such as those based on SiC and Si_3_N_4_ is the polymer derived ceramics (PDCs) route [9,10,11,12,13,14,15,16,17,18,19]. Such a method uses preformed preceramic polymers to be modified at molecular scale to form the nanocomposite precursors. The latter offers the advantages for the in-situ synthesis of the ceramic nano-precipitates in a ceramic (possibility of different nature) matrix during the pyrolysis and annealing experiments. In addition, it allows processing materials in particular shapes and morphologies (dense or porous) that are difficult, or even impossible to obtain from conventional routes [20,21,22,23,24,25].

**Figure 1:**
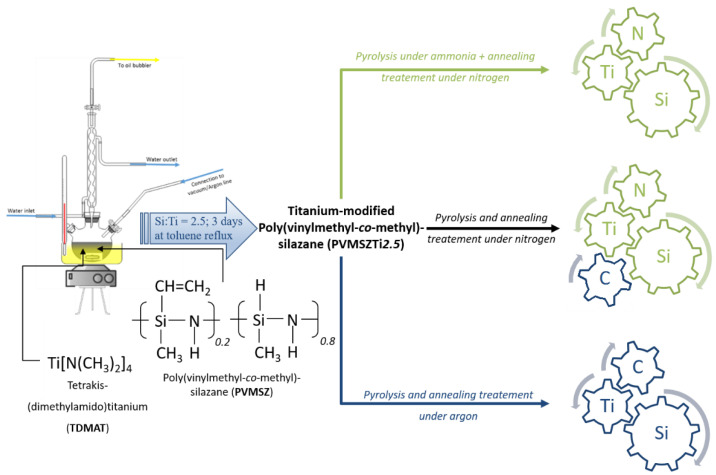
Schematic diagram of the general process of design of nanocomposites in the Si-Ti-N and Si-Ti-C systems from poly(vinylmethyl-*co*-methyl)silazane.

In the present paper, we investigate the PDCs route to design titanium nitride/silicon nitride (TiN/Si_3_N_4_) and titanium carbide/silicon carbide (TiC/SiC) nanocomposites using a commercial liquid polysilazane. The preparation of such nanocomposites usually involves the use of polysilazanes (TiN/Si_3_N_4_) [26,27,28] and polycarbosilanes (TiC/SiC) [5,29,30] as preformed preceramic polymers. Herein, our approach involves first the synthesis of the nanocomposite precursor containing Si, Ti, C, N and H elements from a poly(vinylmethyl-*co*-methyl)silazane which has been modified upon reaction with tetrakis(dimethylamido)titanium. The thermo-chemical behavior of this precursor under various atmospheres is directed to tailor the structure of the final materials: the polymer is converted by heat-treatments (=pyrolysis) at 1000 °C under ammonia, nitrogen or argon into single-phase amorphous ceramics with adjusted compositions; in particular their C and N contents. The latter are subsequently annealed at higher temperatures in flowing nitrogen or argon to initiate the crystallization of the nanophase, i.e., TiN, TiC_x_N_1-x_ or TiC, and then of the matrix, i.e., Si_3_N_4_ or SiC. Thus, this approach provides the material with tuned phase composition and nano-/microstructure organization according to the temperature of annealing and the nature of the atmosphere applied during the pyrolysis. To build further knowledge toward a more rational approach to the preparation of ceramic nanocomposites in nitride, carbonitride and carbide systems—Si-Ti-N (TiN/Si_3_N_4_), Si-Ti-N-C (TiN_x_C_y_ (x + y = 1)/SiC), Si-Ti-C (TiC/SiC)—as reported in the schematic representation of the synthetic process in Figure 1, the present work aims at (i) investigating the structure of a novel titanium-modified polysilazane, (ii) providing a comprehensive mechanistic study of the polymer-to-amorphous ceramic conversion during the pyrolysis of the precursor at 1000 °C in flowing ammonia, nitrogen and argon and (iii) characterizing the high temperature behavior of single-phase amorphous ceramics during their annealing and transformation into the titled nanocomposites. Thus, the results allow for a knowledge-based preparative path toward nanostructured polymer-derived ceramics with adjusted compositions and microstructures.

## 2. Results and Discussion

### 2.1. Polymer Synthesis and Characterization

The titanium-modified polysilazane labeled PVMSZTi*2.5* has been synthesized via the reaction between tetrakis(dimethylamido)titanium Ti[N(CH_3_)_2_]_4_ (TDMAT as titanium source) and a commercially available poly(vinylmethyl-*co*-methyl)silazane labeled PVMSZ by fixing a PMVSZ (monomeric unit):TDMAT ratio of 2.5.

The modification of polysilazanes with TDMAT relies in general on the reaction between the methyl-/dimethylamino groups of TDMAT and the silicon centers of PVMSZ, causing the decrease in the number of Si–NH–Si (Equation (1)) and Si–H (Equation (2)) groups in the obtained precursors and the concomitant evolution of dimethylamine and methane during the synthesis [26,27]. It should be noted that TDMAT can also react with NH groups from the monomeric unit (Si(CH_3_)(CH=CH_2_)NH)_0.2_). Moreover, as-formed -N-Ti[N(CH_3_)_2_]_3_ groups and -Si-N(CH_3_)-Ti[N(CH_3_)_2_]_3_ units can subsequently condense with free SiH and/or NH units or stay as they stand. To support this discussion, we have characterized the precursor by elemental analysis, FTIR and NMR spectroscopies.

-Si-H + H_3_C-N(CH_3_)-Ti[N(CH_3_)_2_]_3_ →-Si-N(CH_3_)-Ti[N(CH_3_)_2_]_3_ + CH_4_ (1)

-N-H + N(CH_3_)_2_-Ti[N(CH_3_)_2_]_3_ →-N-Ti[N(CH_3_)_2_]_3_ + HN(CH_3_)_2_ (2)

Compared to the chemical formula of PVMSZ ([Si_1.0_C_1.5_N_1.1_H_5.5_]_n_ (oxygen content in the sample is 0.4 wt% and can be therefore neglected), the elemental analysis data of the air- and moisture-sensitive solid PVMSZTi*2.5* sample—which allowed to determine a chemical formula of [Si_1.0_Ti_0.3_C_3.0_N_1.6_H_8.5_]_n_ (oxygen content in the sample is 0.6 wt% and can be therefore neglected)—confirms that (i) Ti is incorporated at molecular scale in the structure of PVMSZ and (ii) the carbon, nitrogen and hydrogen contents significantly increase. The measured formula shows that the Si:Ti ratio is higher (i.e., 3.3) than the targeted value fixed before the synthesis, i.e., 2.5, indicating that the Ti content is lower than expected. Most probably, the TDMAT introduced during the polymer synthesis has not completely reacted with PVMSZ. Thus, a certain amount of unreacted TDMAT could be recovered during the extraction of the solvent. Therefore, it is suggested that the reaction cannot be completed when too much TDMAT is added due to a high steric hindrance imposed by the evolutive PVMSZ structure, i.e., the Si:Ti ratio becomes too small and the reactive functions of the neat PVMSZ are no longer accessible. Based on Equations (1) and (2), the significant increase of the carbon, nitrogen and hydrogen contents in the PVMSZTi*2.5* sample can be caused by the formation of Si-N(CH_3_)-Ti bridges. However, this significant increase is most probably related to the high portion of -Ti[N(CH_3_)_2_]_3_ units which are present in as-formed units. They can be considered as side groups that do not further react during the synthesis progress. Thus, this means that the PVMSZTi*2.5* sample displays a higher degree of crosslinking than PVMSZ because of the formation of Ni-Ti bonds and -Si-N(CH_3_)-Ti- bridges and a relative high portion of surrounding-[N(CH_3_)_2_]_3_ groups. In order to obtain a complete view of the titanium-modified polysilazane structure, the PVMSZTi*2.5* sample has been characterized by infrared sand solid-state NMR spectroscopies.

The FTIR spectrum of PVMSZTi*2.5* (Figure S1 in Supplementary Materials) displays some of the characteristic absorption bands of neat PVMSZ [31,32] attributed to the stretching of N-H bonds at 3376 cm^−1^ coupled to N-H deformations at 1174 cm^−1^, the stretching of C-H bonds in CH_x_ groups at 2960 cm^−1^ and the stretching of Si-H bonds and deformations of Si-CH_3_ units at 2123 cm^−1^ and at 1250 cm^−1^, respectively. Below 1056 cm^−1^, absorption bands attributed to the stretching and deformation vibrations involving Si-C, Si-N, C-H, C-C and N-Ti bonds overlap and cannot be assigned unambiguously. The main changes occur in the intensity of some of the characteristic bands quoted above. Thus, a decrease in the intensity of the absorption bands assigned to Si-NH-Si groups at 3376 and 1174 cm^−1^ and Si-H bonds at 2123 cm^−1^ is observed in the spectrum of PVMSZTi*2.5*—more significantly for the bands assigned to N-H bonds. In parallel, there is appearance of a set of broad bands in the range of 2750–2900 cm^−1^ attributed to the vibration of C-H bonds from the methyl/dimethylamino groups present in TDMAT. The band at 1294 cm^−1^ can be assigned to the N-C bonds in -N(CH_3_) groups. Another set of bands that can be attributed to deformation of C-H bands appears at around 1417 and 1457 cm^−1^. Thus, FTIR spectroscopy confirms the incorporation of -N(CH_3_) groups in PVMSZ possibly via Equations (1) and (2):

(i) those involving -NH units in PVMSZ and -N(CH_3_)_2_ groups in TDMAT to form -N-Ti[N(CH_3_)_2_]_3_ units releasing dimethylamine according to Equation (1). This reaction occurs probably majoritarly;
(ii) those involving the silicon centers of PVMSZ and -N(CH_3_)_2_ groups in TDMAT causing the decrease of Si-H groups while forming -Si-N(CH_3_)-Ti- bridges in the obtained precursor and the concomitant evolution of methane according to Equation (2).

Moreover, the band at 3046 cm^−1^, which is assigned to the vibration of C–H bond in the vinyl groups and the typical absorption band arising from the stretching of C=C double bonds in vinyl groups at 1591 cm^−1^ present in PVMSZ are almost vanished in the FTIR spectrum of PVMSZTi*2.5*. It is rather suggested that TDMAT acts as a catalyst for the polymerization of the vinyl groups (Equation (3) and/or the hydrosilylation reaction between -Si–H and -Si-CH=CH_2_ units leading to the formation of carbosilane bonds (-Si-C-C-Si-) (Equation (4)). Indeed, inorganic catalysts such as transition metals or metal complexes could remarkably increase the hydrosilylation rate as well as lower the temperature required for hydrosilylation [33,34,35,36].

*n* -Si-CH=CH_2_ → -(CH(-Si)-CH_2_)_n_ (3)

-Si-CH=CH_2_ + -Si-H → -Si-CH_2_-CH_2_-Si- and/or -Si-CH(CH_3_)-Si- (4)

**Figure 2:**
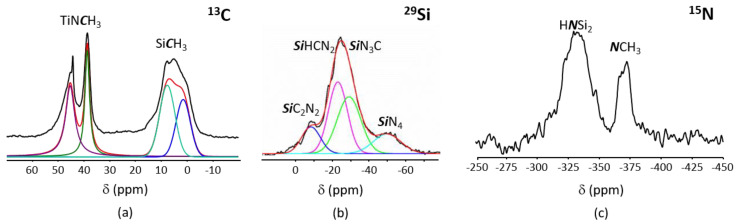
Experimental ^13^C CP MAS NMR (**a**), ^29^Si MAS NMR (**b**) and ^15^N CP MAS NMR (**c**) spectra recorded for the PVMSZTi*2.5* sample at 7 T.

To support this discussion, the FTIR spectra of PVMSZTi*2.5* and a PVMSZ having undergone the same thermal procedure than PVMSZTi*2.5* without TDMAT—it is labeled PVMSZ_T—have been compared (Figure S1 in Supplementary Materials). In the FTIR of PVMSZ_T, the bands attributed to vinyl groups are still present although their intensities decrease. Thus, this confirms the catalytic activity of TDMAT towards reactions involving vinyl groups. These results point out at three or four main effects that took place during the reaction of PVMSZ with TDMAT as depicted in Equations (1) to (4). To achieve an in-depth understanding of the local carbon, silicon and nitrogen environments in the polymer, we investigated ^13^C, ^29^Si and ^15^N solid-state NMR spectroscopy of the PVMSZTi*2.5* sample (Figure 2). The cross-polarization (CP) technique has been used for ^13^C and ^15^N-NMR experiments to obtain spectra with reasonable acquisition times and signal-to-noise ratios.

The solid-state ^13^C CP MAS NMR spectrum of PVMSZTi*2.5* (Figure 2a) exhibits signals which can be simulated with four components as already performed with titanium-modified polymethylsilazane [27]. The signal emerging around 6 ppm is very broad with shoulders present around 0 and 14 ppm. It is deconvoluted into two main components at 1 and 8 ppm to reproduce the shape of the signal although the shoulder at 14 ppm could not be included in this deconvolution. ^13^C-NMR signals at around 0 ppm are typical of carbon atoms of aliphatic groups bonded to a silicon atom [31,32,37,38,39,40,41], i.e., in this case, the silylmethyl group -Si*C*H_3_ as identified in neat PVMSZ. The presence of two signals can be due to the two types of SiCH_3_ unit-containing environments proposed in Equations (1) and (2):

(i) in SiCN_3_ environments (i.e., Si environment after reaction of the SiH groups in the monomeric unit [Si(CH_3_)(H)NH]_0.8_ with TDMAT,
(ii) in SiCRN_2_ (R = H (i.e., Si environment in the monomeric unit [Si(CH_3_)(H)NH]_0.8_) or R = C (i.e., Si environment in the monomeric unit [Si(CH_3_)(CH=CH_2_)NH]_0.2_)); thus units present in PVMSZ that did not react upon chemical modification with TDMAT.

Moreover, this broad signal probably also contains minor signals corresponding to aliphatic carbon atoms directly linked to a silicon as Si-(*C*H-CH_2_)_n_- groups formed as proposed through Equation (3) or Si-*C*H_2_-*C*H_2_-Si and Si-*C*H(CH_3_)-Si units formed by hydrosilylation reactions as proposed through Equation (4). The weak broad signal at around 14 ppm is in the aliphatic region and can be tentatively assigned to aliphatic carbons not directly linked to a silicon such as Si-(CH-*C*H_2_)_n_ or Si-CH(*C*H_3_)-Si groups. The resonances at 39 and 45 ppm are assigned to -TiN*C*H_3_ groups [27]. In particular, the peak at 45 ppm is attributed to N*C*H_3_ groups linked to titanium, i.e., -Si-N(Si-)-Ti[NCH_3_)_2_]_3_ units, resulting from Equation (1). Indeed, its position is similar to the position of the signal identified in the liquid-state ^13^C-NMR spectrum of TDMAT (Figure S2 in Supplementary Materials). Consequently, the signal at 39 ppm is assigned to -N(*C*H_3_) groups in N_2_Si(CH_3_)-N(CH_3_)-Ti[NCH_3_)_2_]_3_ units resulting from Equation (2). It should be mentioned that signals at 124 and 138 ppm—attributed to the carbon of the vinyl groups present in PVMSZ—are identified the PVMSZTi*2.5* sample (Figure S3 in Supplementary Materials), but the weak intensity of the signals confirms that hydrosilylation is almost complete during the synthesis of the PVMSZTi*2.5* sample. The ^29^Si MAS spectrum of the PVMSZTi*2.5* sample (Figure 2b) is composed of a main broad resonance at around −24 ppm that can be fitted with two components at −21 and −29 ppm similarly to titanium-modified polymethylsilazane [27]. The signal at −21 ppm is related to H*Si*CN_2_ environments, i.e., CH_3_-Si(H)N_2_ units as present in PVMSZ [31,32]. The other signal at −29 ppm could correspond to *Si*N_3_C environments and more precisely to N_2_Si(CH_3_)-N(CH_3_)-Ti[NCH_3_)_2_]_3_ units resulting from Equation (2). In addition, there is a signal at around −9 ppm that is attributed to *Si*C_2_N_2_ environments. The ^29^Si chemical shift of SiC_2_N_2_ environments depends on the conformation of the silazane [41]. Thus, the PVMSZTi*2.5* sample is preferentially composed of six- and eight-membered Si-N rings. The signal around −50 ppm is typically for *Si*N_4_ environment although it is difficult to imagine such an environment in our polymeric system. One reason could be the presence of a small portion of ending groups in PVMSZ that react with TDMAT for the existence of such an environment. The experimental ^15^N CP MAS NMR spectrum of the PVMSZTi*2.5* sample (Figure 2c) shows two broad signals centered at −330 ppm and −370 ppm and confirms the previous discussion. The signal at −330 ppm is attributed to H*N*Si_2_ environments since these groups in the silazane backbone are expected between −335 ppm and −325 ppm [31]. The additional signal centered at −370 ppm is assigned to *N*CH_3_ environments based on our data collected for titanium-modified polymethylsilazane [27]. 

The combination of multinuclear solid-state NMR data with results derived from elemental analyses and FTIR allows to have a complete understanding of the chemistry behind the reaction between PVMSZ and TDMAT and a full view of the structure of the PVMSZTi*2.5* sample. At least three reactions depicted in Equations (1) to (4) occur during the synthesis of the PVMSZTi*2.5* sample. They allow building the polymer network and extending the degree of cross-linking of the polymeric backbone. Titanium atoms are homogeneously distributed within the PVMSZ structure as bridges i.e., those involving -(Si-N)_n_-Ti- units (Equation (1)) and those involving N_2_Si(CH_3_)-N(CH_3_)-Ti- units (Equation (2)). In addition, hydrosilylation occurs during the synthesis to form carbosilane bridges (Equation (4)). This leads to a relatively high crosslinked polyme containing a certain portion of side grops of the type -Ti[N(CH_3_)_2_]_x_. The modification of the structure of PVMSZ—because of its reaction with TDMAT—will affect its thermo-chemical behavior. The latter is investigated in the following section through pyrolysis procedures up to 1000 °C in various atmospheres that allowed delivering compounds with controlled phase composition.

### 2.2. Ceramic Conversion

Here, we first discuss the thermo-chemical transformation of the PVMSZTi*2.5* sample into ceramic materials up to 1000 °C in flowing ammonia, nitrogen and argon as monitored by TG experiments. The collection of TG experiments allows having a good overview of the reactivity of the PVMSZTi*2.5* sample with the different types of atmospheres. Then, the polymer-to-ceramic conversion has been investigated in more details using tools complementary to TG experiments: (i) MS to identify the gases evolving from the polymer during TG experiments under argon and nitrogen-MS was not possible to be used in flowing ammonia, (ii) ex-situ spectroscopic analyses (FTIR and/or solid-state NMR) of intermediates isolated during pyrolysis in such atmospheres to follow the evolution of the chemical bonding and environments. All samples produced at 1000 °C have been characterized by solid-state NMR.

#### 2.2.1. TG Experiments

**Figure 3:**
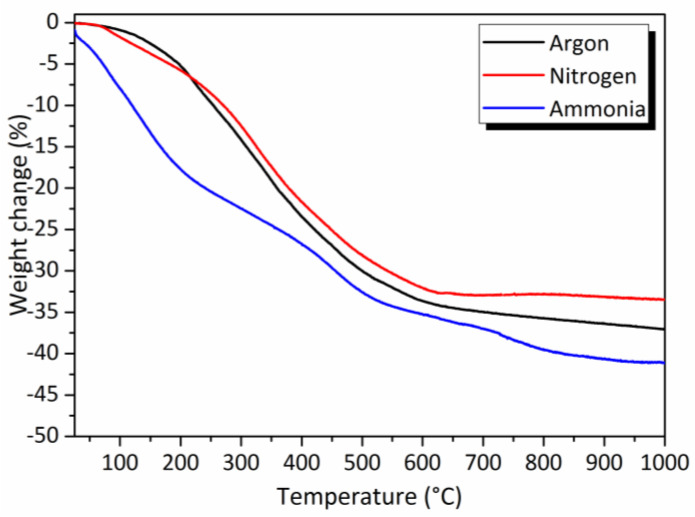
TG curves recorded during decomposition of the PVMSZTi*2.5* sample in flowing ammonia, nitrogen and argon.

The thermo-chemical conversion of preceramic polymers into ceramics occurs through the evolution of gaseous by-products involving a weight loss upon the heat treatment as reported in Figure 3. Results are compared to data recorded from PMVSZ (Figure S4 in Supplementary Materials). In contrast to TG curves recorded in nitrogen and argon atmospheres that exhibit one single-step weight loss which is almost achieved at 700 °C, the TG curve of the PVMSZTi*2.5* sample recorded in an ammonia atmosphere is more complex and displays a three-step weight loss similarly to PVMSZ (Figure S4 in Supplementary Materials) in the temperature range 30–1000 °C, i.e., from 30 to 250 °C (1st weight loss), from 250 to 550 °C (2nd weight loss) and from 550 to 1000 °C (3rd weight loss).

The PVMSZTi*2.5* sample is decomposed with weight losses measured at 1000 °C higher than those measured for PMVSZ (Figure S4 in Supplementary Materials) in the same atmospheres whereas the degree of crosslinking in PVMSZTi*2.5* is supposed to be higher than in PVMSZ. As-formed units are indeed expected to reduce the segment mobility of PVMSZ hindering depolymerization reactions in the polymeric network in the low temperature regime of the polymer-to-ceramic conversion; thereby reducing the total weight loss of the precursor. However, in the present case, we observe that a high cross-linking is not the only prerequisite to design preceramic polymers with high and optimized ceramic yield. Another important issue is a sufficient latent reactivity of the precursors, i.e., the ability to undergo further cross-linking reactions during the heat treatment. This is particularly the case when -Si-H, -N-H and vinyl groups are present in the polymer to occur dehydrocoupling and hydrosilylation reactions. Such units are supposed to be in a significant lower portion in PVMSZTi*2.5* compared to PVMSZ because they reacted with TDMAT during its synthesis. Furthermore, TDMAT catalyzed hydrosilylation reactions during the PVMSZTi*2.5* synthesis. Therefore, dehydrocoupling and hydrosilylation reactions are limited during the heat-treatment of the PVMSZTi*2.5* sample. Finally, the highest weight losses recorded for PVMSZTi*2.5* is also a consequence of the presence of -N(CH_3_)_3_ as side groups in its structure. They are not able to undergo cross-linking reactions and are decomposed during the polymer-to-ceramic conversion. This is particularly understandable in flowing ammonia because -N[(CH_3_)_x_]_y_ units (x = 1 or 2 and y = 1 (for x = 1) and 1 → 3 (for x = 2)) are highly reactive with ammonia to release amines as gaseous by-products via transamination reactions as illustrated in Equation (5).

−Ti[N(CH_3_)_x_]_y_ + yNH_3_ (g) → -Ti[NH_2_]_y_ + y(CH_3_)_x_NH_3−x_ (5)

Since the polymer-to-ceramic conversion of preceramic polymers into ceramic materials is a complex process, spectroscopic analyses (MS, FTIR and/or solid-state NMR) must be very extensive. Thus, detailed discussions of these efforts are reported in detail hereafter.

#### 2.2.2. Pyrolysis in Flowing Ammonia

**Figure 4:**
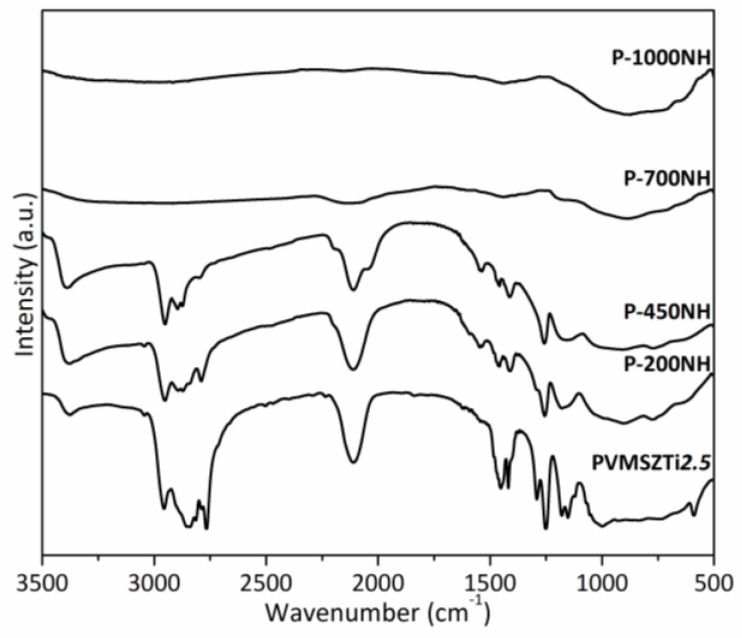
FTIR spectra of pyrolysis intermediates isolated in flowing ammonia and derived from PVMSZTi*2.5*.

To elucidate the mechanisms governing the polymer-to-ceramic conversion in flowing ammonia, intermediates have been isolated during the pyrolysis of PVMSZTi*2.5* at 200, 450, 700 and 1000 °C with a dwelling time of 2 h at each temperature. Pyrolysis intermediates are labeled **P-TNH** (T being the temperature at which the sample was exposed; NH corresponds to ammonia atmosphere). Representative FTIR has been first carried out. The corresponding spectra are shown in Figure 4.

The **P-200NH** sample spectrum displays the characteristic absorption bands of the PVMSZTi*2.5* sample with a main decrease in intensity of the C-H (from methylamino groups) vibration and deformation bands from 2750 to 2900 cm^−1^ and from 1375 to 1500 cm^−1^. These changes are confirmed in the spectrum of the **P-450NH** sample. Thus, these observations prove the occurrence of the reaction depicted in Equation (5) in the low temperature regime of the pyrolysis (T < 450 °C) as suggested based on TG experiments. To support our observations, the N-H vibration band around 3380 cm^−1^ becomes much broader toward lower wavenumbers and more intense suggesting the formation of both -NH and-NH_2_ units as expected through Equation (5). This can be explained by the fact that -Ti[NH_2_]_y_ groups formed in Equation (5) can further condense with formation of NH-containing bridges such as Ti-NH-Ti bridges by release of ammonia according to Equation (6).

-Ti-NH_2_ + -Ti-NH_2_ → -Ti-NH-Ti- + NH_3_ (g) (6)

In contrast, the intensities of the Si-H vibration band around 2120 cm^−1^ and SiCH_3_ deformation band around 1250 cm^−1^ are relatively stable in the corresponding spectra indicating their poor reactivity under ammonia below 450 °C. Changes occur in the spectrum of the **P-700NH** sample. The intensity of the bands attributed to these units significantly decrease demonstrating that Si-H and Si-CH_3_ groups (as well as Si-CH_2_-CH_2_-Si/Si-CH(CH_3_)-Si units) react with ammonia at intermediate temperatures. It is—in general—reported in the temperature range 400–700 °C for polysilazanes [42,43] according to a nucleophilic substitution by ammonia that releases dihydrogen and methane (Equation (7)).

-Si-R + NH_3_ → -Si-NH_2_ +RH (R = H, CH_3_, C_2_H_4_Si-) (7)

As proposed by Choong Kwet Yive et al. for polysilazanes [42,43], homolytic cleavages are the most probable reactions occurring in a parallel way according to Equations (8)–(10).

-Si-R → -Si^•^ + R^•^ (R = H, CH_3_, C_2_H_4_Si-) (8)

R^•^ + NH_3_ (g) → RH + ^•^NH_2_ (9)

-Si^•^ + ^•^NH_2_ → -SiNH_2_ (10)

As-formed -SiNH_2_ groups could self-condense at intermediate temperature (Equation (11)) and in the highest temperature regime of the pyrolysis (T > 700 °C, Equation (12)) during the conversion process with formation of Si-NH-Si and -N(Si)_3_ units by release of ammonia.

-Si-NH_2_ + -Si-NH_2_ → -Si-NH-Si- + NH_3_ (11)

-Si-NH-Si- + -Si-NH_2_ → -N(Si)_3_ + NH_3_ (12)

Similarly, -Si-NH_2_ groups (Equation (11)) as well as -TiNH-Ti- units (Equation (6)) can condense with -TiNH_2_ (Equation (5)) to form -Si-NH-Ti- and -N(Ti)_3_ units, respectively. In parallel, we cannot exclude that unreacted SiH and NH units can react together at high temperature to release hydrogen. Thus, the decomposition of the majority of functional groups is observed in the **P-700NH** sample to form -Si-N- and -Ti-N- bonds. In addition to this observation, the alteration in the shape of the absorption bands compared to the earlier state is due to the amorphous character of the material. This is confirmed with the broadness of the main signal in the spectrum recorded after pyrolysis at 1000 °C (**P-1000NH**). There are no more organic groups and the spectrum is expected to exhibit a mixture of ‘SiN’ and ‘TiN’ units as discussed earlier.

**Figure 5:**
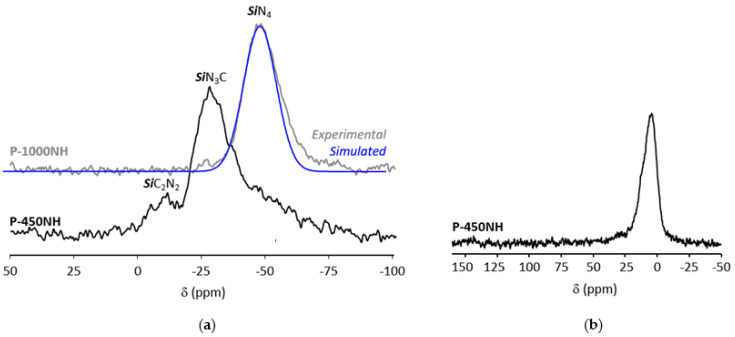
Experimental ^29^Si MAS NMR (**a**) and ^13^C CP MAS NMR (**b**) spectra recorded for the pyrolysis intermediates derived from PVMSZTi*2.5* at 7 T.

Multinuclear solid-state NMR spectroscopy (Figure 5) has been used to identify the most important structural rearrangements occurring during the thermo-chemical conversion of PVMSZTi*2.5* in flowing ammonia. Within this context, the pyrolysis intermediates isolated at 450 °C (**P-450NH**) and 1000 °C (**P-1000NH**) have been analyzed by probing the local environment around silicon (^29^Si, Figure 5a) and carbon (^13^C, Figure 5b).

**Figure 6:**
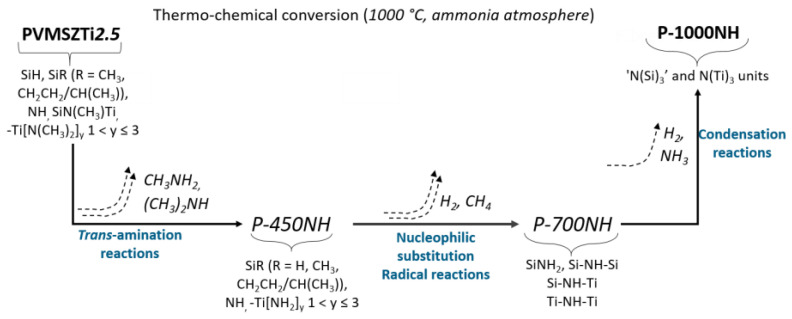
Schematic diagram of the thermo-chemical conversion of titanium-modified poly(vinylmethyl-*co*-methyl)silazanes into Si-Ti-N ceramics in flowing ammonia.

Compared to the ^29^Si MAS spectrum of the PVMSZTi*2.5* sample (Figure 2b), the ^29^Si MAS spectrum of the **P-450NH** sample (Figure 5a) results in a small shift of the main signal toward the resonance at −28 ppm attributed to *Si*N_3_C units while the signal assigned to *Si*C_2_N_2_ units is retained. This suggests that H***Si***CN_2_ units (and *Si*N_4_ units) are the main environment affected by the pyrolysis under ammonia in the low temperature regime of the pyrolysis. In parallel, the ^13^C CP MAS signals at 39 and 45 ppm in the spectrum of the **P-450NH** sample attributed to -TiN*C*H_3_ units disappear (Figure 5b) compared to the ^13^C CP MAS spectrum of the **PVMSZTi*2.5*** sample (Figure 2a). This confirms that such units are decomposed during the first step of the pyrolysis under ammonia as suggested by FTIR. Thus, the spectrum of the **P-450NH** sample shows one main signal around 10 ppm assigned to carbon atoms of aliphatic groups bonded to a silicon atom and/or a carbon atom linked to another aliphatic carbon. The presence of Si-CH(Si-)-*C*H_3_ units or Si-(*C*H-CH_2_)_n_ environments confirms that such units persist upon further heat-treatment at intermediate temperature in flowing ammonia. More changes take place in the **P-1000NH** sample. Obviously, nucleophilic substitutions and rearrangements occur in the temperature range 450–1000 °C that cause a modification of the chemical environment around the silicon atoms. Solid-state NMR suggests a predominantly covalent material in which silicon is mainly present in *Si*N_4_ environments (the corresponding signal can be simulated with one component) confirming the occurrence of reactions depicted from Equations (7) and (12). Thus, based on FTIR and NMR spectroscopies and deduced Equations, the **P-1000NH** sample is made of -N(Si)_3_ and -N(Ti)_3_ units. As a consequence, the thermo-chemical conversion of the PVMSZTi*2.5* sample in flowing ammonia can be seen as represented in Figure 6.

#### 2.2.3. Pyrolysis in Flowing Nitrogen and Argon

- Pyrolysis in flowing nitrogen

**Figure 7:**
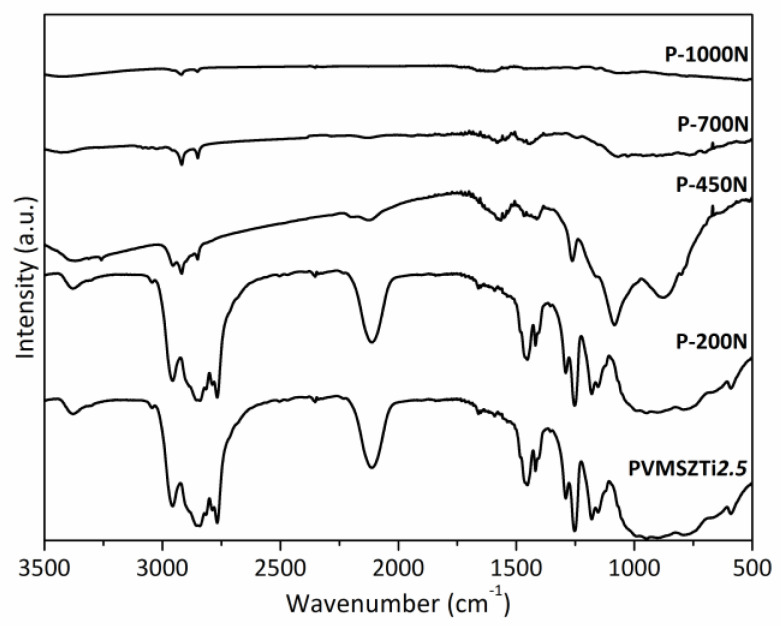
FTIR spectra of pyrolysis intermediates isolated in flowing nitrogen and derived from PVMSZTi*2.5*.

Pyrolysis intermediates have been also isolated under nitrogen during the pyrolysis of PVMSZTi*2.5* at 200, 450, 700 and 1000 °C with a dwelling time of 2 h at each temperature. Pyrolysis intermediates are labeled **P-TN** (T being the temperature at which the sample was exposed; N corresponds to nitrogen atmosphere). Representative FTIR has been first carried out. The corresponding spectra are shown in Figure 7. 

**Figure 8:**
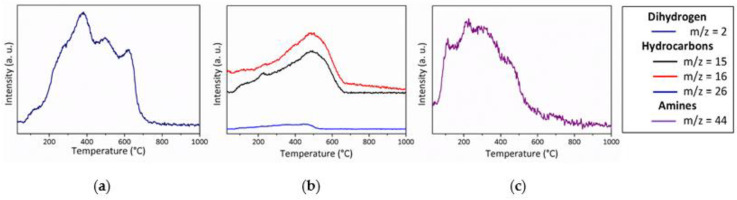
MS curves recorded during TG experiments of the PVMSZTi*2.5* sample in flowing nitrogen: dihydrogen (**a**), hydrocarbons (**b**) and amines (**c**).

In good agreement with the relative stability of the PVMSZTi*2.5* sample during TG experiments up to 200 °C (around 6 wt% of weight loss to be compared to around 17 wt% in flowing ammonia at the same temperature), the **P-200N** sample spectrum displays the characteristic absorption bands of the PVMSZTi*2.5* sample with a relative stability in terms of intensity of bands attributed to the bonds characterizing the functional groups such as C-H, N-H and Si-H. To corroborate this observation, we investigated MS during the TG experiment in flowing nitrogen (Figure 8).

The MS curves show that the temperature range R.T. 200 °C is only associated with a release of dimethylamine (*m*/*z* = 44) fragments indicating the occurrence of condensation reactions involving side groups of the type -N((CH_3_)_2_)_y_ (y = 1 → 3) and NH units which already took place during the synthesis of the PVMSZTi*2.5* sample (Equation (1)). Although the methylamine fragments could not be detected, the release of this gas, however, cannot be excluded in the low temperature regime of the thermo-chemical conversion through the cleavage of the -Si-N(CH_3_)-Ti units formed by Equation (2), then abstracting hydrogen atoms. In this temperature range, MS also identifies the release of dihydrogen (*m*/*z* = 2), most probably because of dehydrocoupling reactions involving NH and SiH groups that did not react during the synthesis of the polymer (-Si-H + -Si-H → -Si-Si- + H_2_ and -N-H + -Si-H → -N-Si- + H_2_). These reactions are well known to occur at relatively low temperature [43] and methane (*m*/*z* = 15, 16) as a continuity of Equation (2). It should be pointed out that these signals can be also attributed to ammonia because of the occurrence of transamination reactions as depicted in Equations (13)–(15) [44].

2 -Si-NH-Si- → -Si-NH_2_ + -N(Si)_3_ (13)

-Si-NH_2_ + -Si-NH-Si- → -N(Si)_3_ + NH_3_ (g) (14)

3-Si-NH-Si- → 2-N(Si)_3_ + NH_3_ (g) (15)

The release of dihydrogen and methane—in addition to acetylene, ethylene or ethane fragments (*m*/*z* = 26)—becomes much more intense above 200 °C in excellent agreement with the significant band intensity changes occurring in the spectrum of the **P-450N** sample: the band intensity of the bonds characterizing the functional groups significantly decreases indicating that the polymer-to-ceramic conversion mainly takes place between 200 and 450 °C as suggested through the TG experiment that identified a weight loss of around 25 wt% at 450 °C. Associated with the main release of dihydrogen and methane, this band intensity decrease continues between 450 and 700 °C (**P-700N**); temperature at which the weight loss (32.5 wt%) is almost complete as indicated through the corresponding TG curve. Only the vibration bands attributed to C-H bond are still present as functional groups. Thus, from 200 to 750 °C, the decomposition reactions involve—in part—radical mechanisms [45,46,47]. As observed with FTIR spectra recorder in flowing ammonia, the absorption bands shape is characteristic of the amorphous character of the material after pyrolysis at 1000 °C (**P-1000N**). They are representative of amorphous ceramics showing no more organic groups and exhibiting a mixture of ‘SiN’, ‘SiC’ units probably also with ‘TiC_x_N_y_’ units (x + y = 1). Hence, based on these results, it seems that the decomposition of the PVMSZTi*2.5* sample under nitrogen first mainly occurs via the polymerization mechanisms depicted in Equations (1) and (2) to crosslink the polymer backbone. Then, radical reactions (Equation (16)) followed by hydrogen abstraction (Equation (17)) take place at intermediate temperature to consolidate the ceramic structure already at 700 °C through the formation of dihydrogen and hydrocarbons.

-Si-R → -Si^•^ + R^•^ (R = H, -CH_3_, -CH_2_-CH_2_-Si-/-CH(CH_3_)Si-) (16)

R^•^ + H^•^→ RH (R = H, -CH_3_, -CH_2_-CH_2_-Si-/-CH(CH_3_)Si-) (17)

Hydrogen radicals are formed by the decomposition of C-H bonds in the high temperature regime of the thermo-chemical conversion. It should be pointed out that the identification of methane can be due to the cleavage of C-C bonds as suggested through Equation (18). Then, the CH_3_^•^ radical can abstract hydrogen from Si-H bonds (Equation (19)).

-Si-CH(CH_3_)-Si → -Si-CH^•^-Si + CH_3_^•^ (18)

CH_3_^•^ + -SiH → -CH_4_ + Si^•^ (19)

The large escape of ethylene CH_2_=CH_2_ may arise from the degradation of the Si-CH_2_-CH_2_-Si units formed by hydrosilation during the synthesis of the PVMSZTi*2.5* sample according to Equations (20) and (21).

Si-CH_2_-CH_2_-Si → -Si^•^ + ^•^CH_2_-CH_2_-Si (20)

^•^CH_2_-CH_2_-Si → Si^•^ + CH_2_ = CH_2_ (21)

The temperature range 700–1000 °C is probably associated with dihydrogen release due to homolytic cleavage of residual C-H bonds followed by H abstraction (Equations (22) and (23)).

-C-H → C^•^ + H^•^ (22)

H^•^ + H^•^ → H_2_ (23)

- Pyrolysis in flowing argon

**Figure 9:**
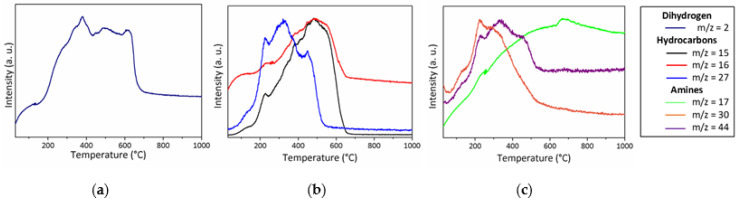
MS curves recorded during TG experiments of the PVMSZTi*2.5* sample in flowing argon: dihydrogen (**a**), hydrocarbons (**b**) and amines (**c**).

The thermo-chemical conversion of the PVMSZTi*2.5* sample in an argon atmosphere is similar to that one observed in flowing nitrogen based on the profile of the weight loss curve recorded during the TG experiments. The main difference takes place in the high temperature regime of the TG experiment since the weight loss is not stabilized after pyrolysis at 1000 °C in flowing argon. To understand the behavior of the PVMSZTi*2.5* sample in an argon atmosphere, in-situ MS experiments were performed and the corresponding curves are represented in Figure 9. 

The nature of the gases that are released from the PVMSZTi*2.5* sample in flowing argon and the temperature ranges in which their release significantly occurs matches with the data recorded in nitrogen atmosphere. Only the fragments at *m*/*z* = 26, 27 and 30 are identified in either nitrogen (*m*/*z* = 26) or argon (*m*/*z* = 27 and 30). The ion signals at *m*/*z* = 26 and 27 most probably correspond to the C_2_H_2_^+^ and C_2_H_3_^+^ fragments; thereby arising due to the same molecules, i.e., ethane and ethylene evolving in both argon and nitrogen. The ion signal at *m*/*z* = 30 corresponds to the release of methylamine that takes place under argon at low temperature as dimethylamine release occurs. The release of methylamine is suggested in flowing nitrogen despite the fact that it is not detected by MS. Thus, the discussion on the thermo-chemical conversion of the PVMSZTi*2.5* sample in flowing argon fits the one given previously under nitrogen and the same mechanisms occur in both atmospheres in similar temperature range. Based on the results from FTIR and MS obtained in both nitrogen and argon atmospheres, it is suggested that the thermo-chemical conversion involves three main steps:

(1) Below 300 °C, the precursor undergoes thermal cross-linking via condensation reactions involving both side groups of the type -N((CH_3_)_2_)_x_ (x = 0, 1, 2, 3) and NH units (Equation (1)) and -Si-N(CH_3_)-Ti- that cleaves upon heating to abstract hydrogen releasing methylamine.
(2) From 300 to 700 °C, mainly hydrocarbons such as methane and ethane/ethylene and hydrogen are released from the evolving system via radical reactions then H abstraction.
(3) Above 700 °C, the remaining hydrogen atoms are gradually removed through homolytic cleavage of C-H bonds and then H abstraction to lead to a single-phase amorphous covalently bonded ceramic.

**Figure 10:**
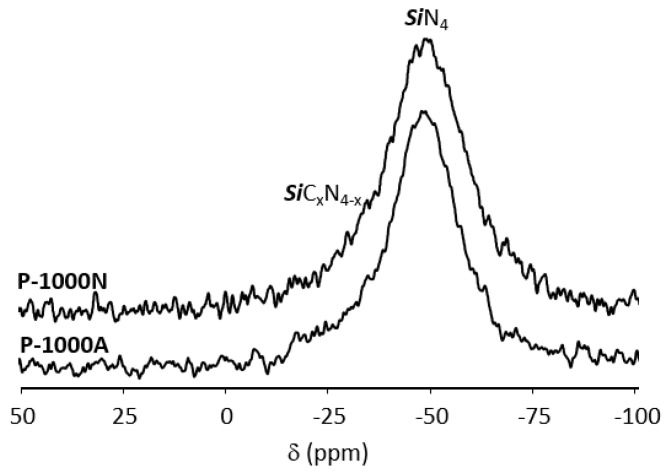
Experimental ^29^Si MAS NMR for the pyrolysis samples isolated at 1000 °C in flowing argon and nitrogen at 7 T.

As reported in Figure 10, these reactions lead to **P-1000N** and **P-1000A** samples both displaying in their ^29^Si MAS spectra a relatively broad signal at around −48 ppm ascribed to *Si*N_4_ environments. However, its broadness—especially on the left part of the main signal—tends to indicate an enrichment in carbon around silicon to form *Si*C_x_N_4-x_ (0 ≤ x ≤ 4) type units compared to the sharper signal identified in the spectrum of the **P-1000NH** sample that can be simulated with one component as indicated in Figure 5.

**Figure 11:**
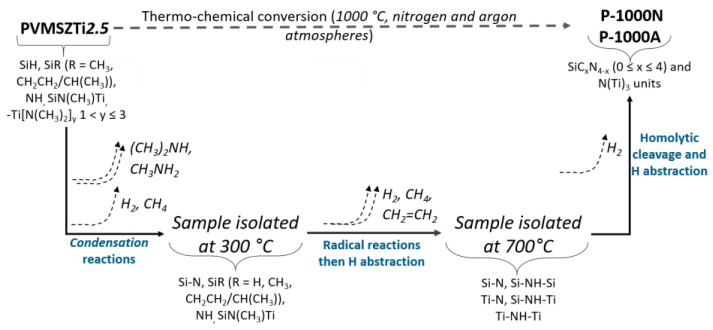
Schematic diagram of the thermo-chemical conversion of titanium-modified poly(vinylmethyl-*co*-methyl)silazanes into Si-Ti-C-N ceramics in flowing nitrogen and argon.

Thus, the thermo-chemical conversion of the PVMSZTi*2.5* sample in flowing nitrogen and argon can be seen as represented in Figure 11 based on the TG data coupled with in-situ MS and ex-situ FTIR and solid-state spectroscopies of pyrolysis intermediates.

### 2.3. High-Temperature Phase and Microstructure Evolution of Single-Phase Amorphous Ceramics

**Figure 12:**
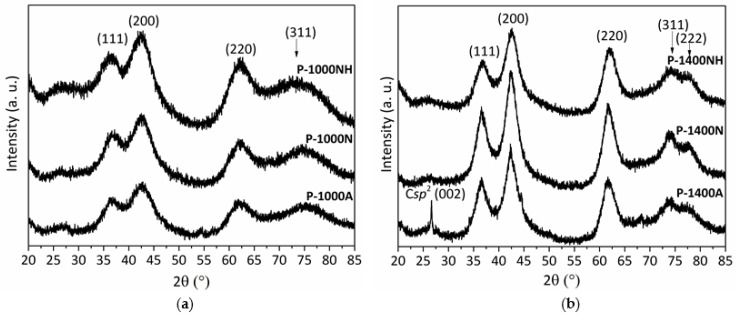
XRD patterns of **P-1000NH**, **P-1000N** and **P-1000A** (**a**) and P-1400NH, P-1400N and the P-1400A (**b**).

After pyrolysis experiments, the X-ray diffractograms reveal that as-pyrolyzed samples are poorly crystallized as shown by the diffuse peaks identified in the patterns of the **P-1000NH**, **P-1000N**, **P-1000A** samples (Figure 12a). In a first approximation, they tend to reveal the slow nucleation of face-centered cubic (*fcc)* TiN crystals (powder diffraction file (ICDD PDF) number: 00-038-1420) according to the presence of peaks at 2θ around 36.5°, 42.6° and 62.3 which can be attributed to the (111), (200) and (220) TiN reflections, respectively. To support this observation, the very broad peak in the range 72.8–75.2° (peak positions changes according to the nature of the atmosphere) could be attributed to the (311) TiN reflection. The crystallite sizes are low and range from 1.4 (**P-1000NH**) to 1.7 nm (**P-1000A**).

For all samples prepared at 1000 °C, EDS analyses demonstrate that:

(i) the initial Si:Ti ratio (2.5) fixed in the polymer is retained in the derived ceramics,
(ii) a strong tendency to decrease the carbon content from the sample treated under argon (**P-1000A**, carbon content of 16.4 wt%) to samples treated under ammonia (**P-1000NH**, carbon content of 1.5 wt%).

Thus, we can suggest that the **P-1000NH** sample is only made of Si, Ti and N whereas the **P-1000N** and **P-1000A** samples are composed of Si, Ti, C, N. The increase of the annealing temperature to 1400 °C under nitrogen (**P-1400NH** and **P-1400N** samples) and argon (**P-1400A** sample) with a dwelling time of 2 h at 1400 °C does not change the solid-state ^29^Si NMR response: all spectra report the broad peak characteristic of *Si*N_4_ environments with—for the **P-1400N** and **P-1400A** samples—emerging peaks in the range −10 to −35 ppm due to *Si*C_4_ and *Si*C_x_N_4-x_ (0 ≤ x ≤ 4) type units (see Figure S5 in Supplementary Materials). *Si*C_4_ environments can be identified in such spectra by comparing them with the spectrum of the **P-1800A** sample which exhibits only this signal. Although solid-state NMR does not show any changes in the chemical environment of Si, XRD investigations show that annealing to 1400 °C involves further crystallization of the materials (Figure 12b). XRD patterns of the **P-1400NH**, **P-1400N**, **P-1400A** samples reveal better defined and shaper peaks at 2θ = 36.5° (**P-1400N** and **P-1400A**) and 36.7° (**P-1400NH**), from 42.3° (**P-1400A**) to 42.6° (**P-1400NH**), from 61.7° (**P-1400A**) to 62.0° (**P-1400NH**), 74.1° and 77.5° (all samples). Since the positions tend to slightly change between samples according to the used atmosphere, we tentatively suggested that the XRD patterns exhibit the (111), (200), (220), (311) and (222) peaks from a TiC_x_N_y_ (x + y = 1) crystalline phase with different stoichiometries. For instance, the peaks (111), (200) and (220) can be indexed according to the TiC_0.3_N_0.7_ phase (ICDD PDF 00-042-1488) at 36.45°, 42.35° and 61.44°; the TiC phase (ICDD PDF 00-032-1383) at 35.90°, 41.70° and 60.44°; and the TiN phase (ICDD PDF 00-038-1420) at 36.66°, 42.59° and 61.81°, diffraction patterns, respectively.

As previously reported using the lattice parameter values for pure TiN and TiC and the lattice parameters of various TiC_x_N_y_ phases, the lattice constant of TiC_x_N_y_ (x + y = 1) is a linear function of its chemical composition which obeys Vegard’s law [48,49]. Thus, the observed shift of the XRD (200) reflection in Figure 12b was used to estimate the chemical composition of the TiC_x_N_y_ (x + y = 1) phase in the ceramics prepared at 1400 °C (Table 1) [50].

No further XRD peaks are observed except the (002) peak attributable to sp^2^ carbon identified in the **P-1400A** sample. Based on the crystallite size (from 1.9 nm (**P-1400NH**) to 3.1 nm (**P-1400N**/**P-1400A**)), the **P-1400NH** sample can be considered as a TiN-based ceramic displaying the lowest crystallization degree.

**Figure 13:**
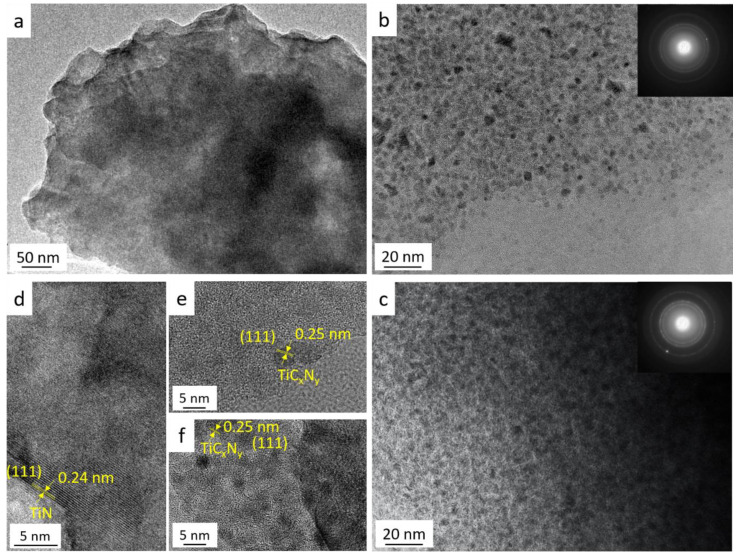
TEM images of P-1400NH (**a**,**d**), P-1400N (**b**,**e**) and the P-1400A samples (**c**,**f**) with inserted selected area electron diffraction patterns in (**a**–**c**).

To assess the phase micro-/nanostructure of these materials, the **P-1400NH**, **P-1400N**, **P-1400A** samples have been further studied by means of TEM (Figure 13). Figure 13a depicts a featureless low magnification TEM image of **P-1400NH** which is significantly different from images recorded for **P-1400N** and **P-1400A** samples. In the latter, specimens consist of homogeneously dispersed small nuclei (dark contrast) embedded in an amorphous network. The Selected Area Electron Diffraction (SAED) patterns of the **P-1400N** and **P-1400A** samples identify more distinct rings which can be indexed to the (111), (200) and (220) plans of the TiC_x_N_y_ phase indicating an extent of the crystallization in those samples. In the high-resolution micrographs (Figure 13d–f), the analysis of the **P-1400NH** sample (Figure 13d) reveals the presence of nanocrystals with a lattice spacing of 0.24 nm corresponding to the *d*-spacing of the lattice plane of the TiN structure, i.e., the (111) direction of the *fcc* cubic rocksalt TiN structure which agrees with the phase identified in the X-ray diffractogram of the same sample (Figure 12b). The HRTEM micrographs in Figure 13e,f demonstrate that crystallization occurs in-situ in the **P-1400N** and **P-1400A** samples. Lattice fringes of the TiC_x_N_y_ nanocrystals—with diameter less than 5 nm as better calculated in Figure 13e,f—are observed which confirms that the samples represent nanocomposites with a fringe spacing of 0.25 nm. As a conclusion, heat-treatment above 1400 °C provides access to nanocomposites composed of nano-precipitate homogeneously distributed in an amorphous matrix. Thus, in the following section, we investigated separately ammonia-treated samples and nitrogen/argon-treated samples.

#### 2.3.1. Ammonia-Treated Samples Heat-Treated in the Temperature Range 1500–1800 °C

**Figure 14:**
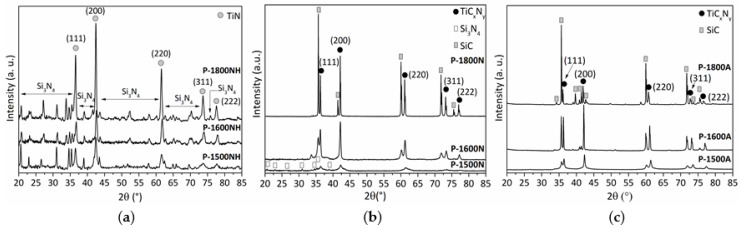
Evolution of the XRD patterns of P-1000NH (**a**), P-1000N (**b**) and P-1000A (**c**) samples in the temperature range 1500–1800 °C.

**Figure 15:**
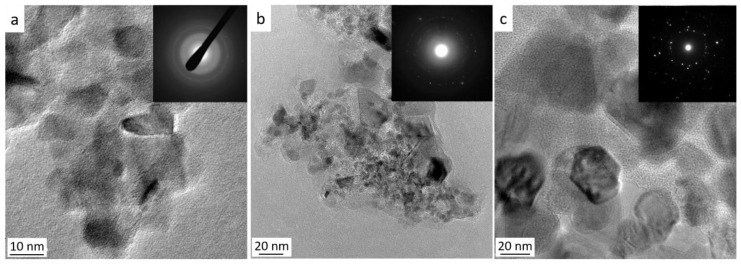
TEM micrographs of **P-1500NH** (**a**), **P-1500N** (**b**) and **P-1500A** (**c**) samples with selected area electron diffraction pattern as insets.

After heat-treatment at 1500 °C, the **P-1500NH** sample shows sharper and more intense TiN XRD peaks (crystallite size = 16 nm) as well as emerging XRD peaks corresponding to nanosized α-Si_3_N_4_ (ICDD PDF number: 04-005-5074) crystals (Figure 14a). HRTEM (Figure 15a) confirms clear evidence for the crystallization process of the TiN nanophase (**P-1500NH**) appearing as small nuclei (around 10 nm). We can clearly distinguish the fcc structure of TiN nanocrystals with a fringe spacing of 0.24 nm (Figure S7a in Supplementary Materials). This is confirmed through the SAED pattern (inset in Figure 15a).

The increase of the annealing temperature confirms TiN crystal growth as shown through the XRD patterns of samples annealed at 1600 °C (**P-1600NH**, crystallite size = 22.4 nm) and 1800 °C (**P-1800NH**, crystallite size = 27.4 nm). Samples represent nanocomposites made of crystallized TiN and Si_3_N_4_ (α and β (ICDD PDF number: 04-033-1160)) phases (Figure 14a). In the following section, we discussed the crystallization behavior of the samples pyrolyzed then annealed in flowing nitrogen and argon together.

#### 2.3.2. Nitrogen and Argon-Treated Samples Heat-Treated in the Temperature Range 1500–1800 °C

**Figure 16:**
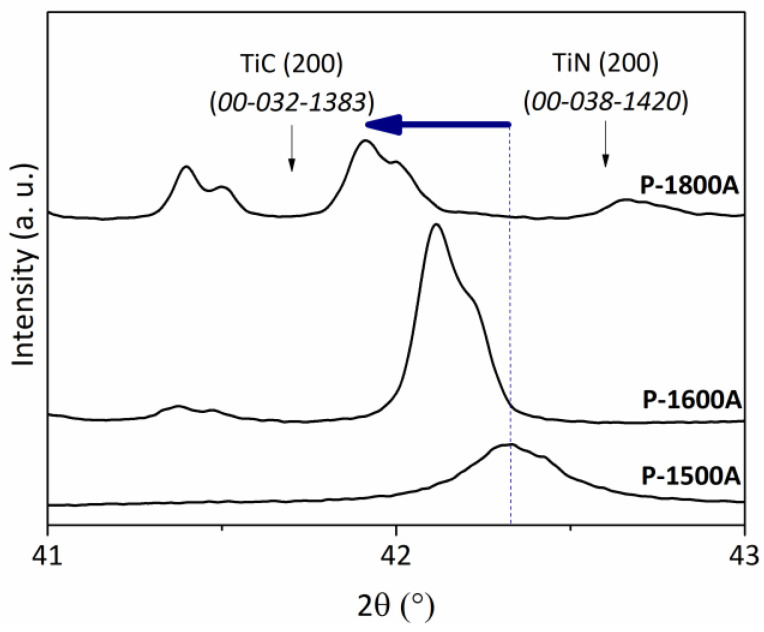
Evolution of the (200) peak position in the X-ray patterns of **P-1500A**, **P-1600A** and **P-1800A** samples.

The increase of the temperature to 1500 °C (**P-1500N** (Figure 14b) and **P-1500A** (Figure 14c)) involves further crystallization of both TiC_0.3_N_0.7_ (crystalline size = 10.8 nm) and α and β-Si_3_N_4_ phases in **P-1500N** (see Figure S6 in Supplementary Materials) and both TiC_0.4_N_0.6_ (crystalline size = 28.4 nm) and β-SiC (ICDD PDF number: 00-029-1129) phases in **P-1500A**. In the latter, we cannot exclude the presence of α/β-Si_3_N_4_ phases because of the relative broadness of the XRD peaks. Thus, compared to the (200) XRD peak position in the XRD pattern of the **P-1400N** and **P-1400A** samples (Figure 12b, Table 1), the reflections of the TiC_x_N_y_ (x + y = 1) phase slightly shift towards lower diffraction angles indicating that the TiC_x_N_y_ (x + y = 1) phase becomes enriched in carbon. This is illustrated in Figure 16 for the **P-1500A** sample and its derivatives at 1600 °C (**P-1600A**) and 1800 °C (**P-1800A**).

Figure 15b,c displays the low magnification images of the **P-1500N** and **P-1500A** samples. They show that nanocrystals with a size that can exceed 20 nm—in particular for the sample formed under argon—co-exist with an amorphous phase appearing as a bright-appearing network. The SAED patterns (insets in Figure 15b,c) are composed of distinct spots that indicate the high degree of crystallinity of TiC_x_N_y_ (x + y = 1) nanocrystals. The high magnification of HRTEM images of both samples exhibit lattice fringe of the TiC_x_N_y_ (x + y = 1) nanophase with an equal fringe spacing of 0.25 nm. (Figure S7 in Supplementary Materials). Pyrolysis temperatures of 1600 °C identified compositions of TiC_0.4_N_0.6_ (**P-1600N**; crystalline size = 25.8 nm) and TiC_0.6_N_0.4_ (**P-1600A**; crystalline size = 43.3 nm) (Table 1) along with SiC. The heat-treatment to 1800 °C involves further crystallization of the phases and we noticed that the diffraction (200) peaks shift toward lower diffraction angles of TiC indicating an increase in the lattice parameter and the formation of the TiC_0.5_N_0.5_ (**P-1800N**; crystalline size = 109.6 nm) and TiC_0.8_N_0.2_ (**P-1800A**; crystalline size = 109.8 nm) solid solutions whereas β-/α-SiC could be assigned to other peaks (Figure 14b,c). From line broadening of the (220) reflection, the β-SiC crystallite size is estimated to be 99.8 nm.

EDS analyses (See EDS mapping in Figures S8 and S9 in Supplementary Materials) of the **P-1600A** and **P-1800A**, samples indicated the gradual formation of TiC/SiC nanocomposites with the increase of the temperature to 1800 °C, especially using argon as an atmosphere. The identification of *Si*C_4_ environments has been confirmed in the **P-1800A** sample via ^29^Si solid-state NMR (See Figure S5 in Supplementary Materials). To be more accurate in the C, N and O contents, we investigated C/S and N/O/H analysis for these samples and, by considering the measurement of Si and Ti contents by EDS, we found that they exhibited chemical formulas of Si_1.0_Ti_0.2_C_0.8_N_0.2_0_0.02_ (**P-1600A**) and Si_1.0_Ti_0.2_C_1.1_N_0.1_0_0.01_ (**P-1800A**) indicating the formation of a nanocomposite composed of 20 wt% of TiC in a SiC matrix free of carbon whereas nitrogen could be neglected according to the very low content (1.5 wt%). Thus, through the modulation of the atmosphere of pyrolysis, we succeeded in the design of nitride, carbonitride and carbide nanocomposites using an unique titanium-modified polysilazane.

## 3. Materials and Methods

### 3.1. General Comments

The synthesis of the precursor is carried out in a purified argon atmosphere passing through a column of phosphorus pentoxide and then a vacuum/argon line by means of standard Schlenk techniques. The cleaned glassware is stored in an oven at 90 °C overnight before being connected to the vacuum/argon line, assembled and pumped under vacuum for 30 min and then filled with argon. All chemical products are handled in an argon-filled glove box (Jacomex, Campus-type; O_2_ and H_2_O concentrations kept at ≤0.1 ppm and ≤0.8 ppm, respectively). Toluene (99.85%, Extra Dry over Molecular Sieve, AcroSeal(R)) was purchased from Acros Organics™. The poly(vinylmethyl-*co*-methyl)silazane (commercial name: Durazane^®^ 1800) was provided by Merck company, Germany, stored in a fridge and used as-received. It is labeled PVMSZ. Anal. Found (wt%): Si, 41.3; C, 27.3; N, 22.7; H, 8.3; O, 0.4. [Si_1.0_C_1.5_N_1.1_H_5.5_]_n_ (Referenced to Si_1.0_ and oxygen content was omitted in the empirical formulae). FTIR (ATR/cm^−1^): ν (N−H) = 3388 (m), ν (C−H) = 3046 (s), 3010 (s), 2954 (s), 2895 (s), 2852 (m), ν (Si−H) = 2121 (vs), δ (vinyl) = 1591 (m), δ (CH_2_) = 1405 (m), δ (Si−CH_3_) = 1251 (s), δ (N-H): 1166 (m), δ (C-H + C-C + N−Si−N + Si-C) = 1005-630 (vs); ^1^H NMR (300 MHz, CDCl_3_, δ/ppm): 0.4–0.1 (br, SiCH_3_), 1.1–0.5 (br, NH), 4.9–4.4 (br, SiH), 6.3–5.7 (br, vinyl). Tetrakis(dimethylamido)titanium (Ti[N(CH_3_)_2_]_4_, 99.99%) was obtained from Acros Organics™, stored in a fridge and used without further purification. It is labeled TDMAT.

### 3.2. Polymer Synthesis

The reaction between PVMSZ and TDMAT is done in toluene at temperature of reflux (115 °C) in a three-neck round-bottom flask. In a typical experiment, 5.7 g of TDMAT (16.5 mmol) is quickly added with a syringe under flowing argon to a solution of 2.7 g of PVMSZ (41.2 mmol referred to the theoretical monomeric unit of the polymer) in 80 mL of toluene at RT under vigorous stirring. Then, the temperature is increased up to 115 °C under static argon and kept at this temperature under vigorous stirring for three days. After cooling down, the solvent is extracted via an ether bridge (100 °C/1.5.10^−1^ mbar) to release an air- and moisture-sensitive titanium-modified PVMSZ labeled PVMSZTi*2.5* (*2.5* being the Si:Ti ratio) that appears as a brownish-black powder. Anal. Found (wt %): Si 25.8, Ti 11.9, C 33.1, N 20.7, H 7.9, O 0.6. [Si_1.0_Ti_0.3_C_3.0_N_1.6_H_8.5_]_n_ (Referenced to Si_1.0_ and oxygen content was omitted in the empirical formulae). FTIR (KBr/cm^−1^): ν(N−H) = 3376 (w), ν (C−H) = 2960 (s), 2848 (s), 2761 (s), ν(Si−H) = 2123 (m), δ(CH_3_) = 1457 (s), δ(CH_2_) = 1417 (m), δ(C-N) = 1294 (m), δ(Si−CH_3_) = 1250 (s), δ (N-H): 1174 (m), δ (C-H + C-C + N−Si−N + Si-C + Si−N−Ti) = 1054-655 (vs).

### 3.3. Synthesis of the Ceramic Materials

Polymeric powders are placed in alumina boats in the glove-box, then introduced in a sealed tube under argon atmosphere to prevent any oxygen contamination of the samples during the transfer to the furnace. Powders are then introduced under argon flow into a silica tube inserted in a horizontal furnace (Carbolite BGHA12/450B). The tube is evacuated (0.1 mbar) and refilled with anhydrous ammonia (99.99%), nitrogen (99.995%) or argon (99.995%) to atmospheric pressure. Subsequently, the samples are subjected to a cycle of ramping of 5 °C.min^−1^ in the temperature range 200–1000 °C in flowing ammonia, nitrogen or argon (dwelling time of 2 h at the temperature selected among 200, 450, 700 and 1000 °C). A constant flow (120 mL min^−1^) is passed through the tube during the pyrolysis cycle. After cooling under argon atmosphere, ammonia-, nitrogen- and argon-pyrolyzed samples are stored in the glove-box for characterization. Samples are labeled P-TX with T being the temperature at which the polymer has been exposed and X corresponding to the nature of the atmosphere: X = A for argon, X = N for nitrogen and X = NH for ammonia. For the high temperature (T > 1000 °C) investigation, samples pyrolyzed at 1000 °C (**P-1000NH**, **P-1000N** and **P-1000A**) are subsequently introduced in a graphite furnace (Gero Model HTK 8 for ammonia-pyrolyzed samples and a Nabertherm VHT-GR for nitrogen- and argon-pyrolyzed samples) for annealing treatments. The furnaces are pumped under vacuum (1.10^−1^ mbar), refilled with nitrogen for ammonia- and nitrogen-pyrolyzed samples or with argon for argon-pyrolyzed samples and maintained under a constant flow of gas (200 mL min^−1^) during the whole heat treatment. The program consists of a 5 °C.min^−1^ heating ramp up to the maximum temperature fixed in the range 1400–1800 °C, dwelling at the selected temperature for 2 h and cooling down to RT at 5 °C.min^−1^. Samples are labeled P-TX with T the temperature at which the polymer has been exposed (1400, 1500, 1600 and 1800 °C) and X corresponds to the nature of the atmosphere: X = A for argon, X = N for nitrogen and X = NH for ammonia (200 °C ≤ T ≤ 1000 °C) and then nitrogen T > 1000 °C.

### 3.4. Material Characterization

As the polymers are reactive towards moisture and oxygen, the following sample preparations were performed within a glove box. The chemical structure of polymers was determined by transmission FTIR spectroscopy using a Nicolet Magna 550 Fourier transform-infrared spectrometer. ^1^H NMR data of PMVSZ solutions in CDCl_3_ were obtained using a Bruker AM 300 spectrometer operating at 300 MHz. Tetramethylsilane (TMS) was used as a reference for the NMR data. Solid-state ^13^C CP MAS, ^15^N CP MAS and ^29^Si MAS NMR spectra were recorded on a Bruker AVANCE 300 spectrometer (7.0 T, ν_0_(^1^H) = 300.29 MHz, ν_0_(^13^C) = 75.51 MHz, ν_0_(^15^N) = 30.44 MHz, ν_0_(^29^Si) = 59.66 MHz) using 4 mm and 7 mm Bruker probes and a spinning frequencies of 10 and 5 kHz, respectively. ^13^C and ^15^N CP MAS experiments were recorded with ramped-amplitude cross-polarization in the ^1^H channel to transfer magnetization from ^1^H to ^13^C and ^15^N. (Recycle delay = 3 s, CP contact time = 1 ms, optimized ^1^H spinal-64 decoupling). Single pulse ^29^Si MAS NMR spectra were recorded with a recycle delay of 60 s. Chemical shift values were referenced to tetramethylsilane for ^13^C and ^29^Si and CH_3_NO_2_ for ^15^N. Chemical analyses of the polymers were performed using a combination of several methods at Mikroanalytisches Labor Pascher (Remagen, Germany). Thermogravimetric analyses (TGA) of samples were performed in flowing ammonia at 5 °C.min^−1^ to 1000 °C using silica crucibles (Setaram TGA 92 16.18, SETARAM Instrumentation (Caluire, France)). In addition, they were done in flowing nitrogen or argon at 5 °C.min^−1^ to 1000 °C using alumina crucibles at ambient atmospheric pressure (STA 449 F3, Netzsch GmbH, Selb, Germany) coupled with mass spectrometer (Omnistar, Pfeiffer Vacuum GmbH, Asslar, Germany) in outlet. The phase composition of ceramic samples was determined by XRD analysis (Bruker AXS D8 Discover, Cu*K*_α_ radiation, Billerica, MA, USA). The scans were performed in the range of 2θ ∈ 〈20°; 85°〉 with a step of 0.015° and an exposure time of 0.7 s. The diffractograms were analyzed using the Diffrac + EVA software with the JCPDS-ICDD database. Crystallite sizes of TiC_x_N_y_ crystals were calculated from the FWHM of the (200) diffraction lines using the Scherrer’s equation while their chemical composition was measured by considering the interlayer spacing *d*_200_ and the lattice parameter *a* considering both the Scherrer equation and the Vegard’s law. The chemical analysis of selected powders was determined by scanning electron microscopy equipped with energy dispersive spectroscope (EDS-SEM, JEOL IT 300 LV, Tokyo, Japan) and confirmed for some of them using hot gas extraction techniques with a Horiba EMGA-830 for oxygen, nitrogen and hydrogen contents and using a combustion analysis method for carbon content on a Horiba Emia-321V. Structural and morphological characterizations were conducted by Transmission Electron Microscopy with a JEM-2100F model from JEOL (Japan) for nitrogen- and argon-treated samples and at the Indian Institute of Technology Madras, Chennai using TECNAI 20G^2^, FEI instruments (Hillsboro, OR, USA) with an accelerating voltage of 200 kV & JEOL 3010 (Japan) at an accelerating voltage of 300 kV for ammonia-treated samples.

## 4. Conclusions

Within the present study, a titanium-modified polysilazane has been synthesized by adding tetrakis(dimethylamido)titanium Ti[N(CH_3_)_2_]_4_ (TDMAT as titanium source) to a commercially available poly(vinylmethyl-*co*-methyl)silazane labeled PVMSZ according to an atomic Si:Ti ratio of 2.5. The effect of the nature of the atmosphere including ammonia, nitrogen and argon behind its thermo-chemical conversion and the crystallization behavior of derived amorphous ceramics has been critically investigated. Synthesis reactions involved N-H and Si-H bonds in polysilazanes and N(CH_3_)-based groups in TDMAT as well as hydrosilylation reactions based on complementary characterization tools including FTIR, solid-state NMR and elemental analysis. Thus, the incorporation of Ti increased the crosslinking degree of the polysilazane and introduced side groups that affect the weight change during the further polymer-to-ceramic conversion. Titanium plays a major role in modifying the mechanisms of the polysilazane-to-ceramic transformation, especially in the low temperature regime of the thermal decomposition by prohibiting the distillation of small polymer fragments occurring with PVMSZ and then releasing amines most probably from side groups and hydrocarbons based on radical reactions. Pyrolysis mechanisms have been identified based on TG experiments, FTIR, solid-state NMR and MS spectroscopies. After pyrolysis to 1000 °C, materials are X-ray amorphous (although nucleation of TiC_x_N_y_ (x + y = 1) nanocrystals could be considered) and display a Si:Ti ratio in good agreement with the ratio fixed the polymers. The final nano-/microstructure evolved according to the annealing temperature fixed in the range 1400–1800 °C. Ammonia-treated amorphous ceramics evolved toward nanocrystalline-TiN/amorphous-Si_3_N_4_ whereas nitrogen- and argon-treated ceramics led to nanocrystalline-TiC_x_N_1-x_/amorphous-SiC(N) nanocomposites after annealing at 1400 °C. At 1800 °C, highly crystallized nanocomposites are obtained in the Si-Ti-N (ammonia-treated ceramics) and Si-Ti-C (argon-treated ceramics) systems. The nitrogen-treated samples represent TiC_x_N_y_ (x + y = 1)/SiC nanocomposites.

## Figures and Tables

**Table 1:** Structural parameters measured based on XRD pattern of nanocomposite samples.

Samples	(200) Peak Position (2θ, °)	d_200_ (nm)	Lattice Parameter a (nm)	Composition of the TiC_x_N_y_ from XRD
**P-1400NH**	42.6	0.212	0.4245	TiN
**P-1400N**	42.45	0.213	0.4264	TiC_0.2_N_0.8_
**P-1400A**	42.3	0.214	0.4273	TiC_0.3_N_0.7_
**P-1500NH**	42.6	0.212	0.4245	TiN
**P-1500N**	42.3	0.214	0.4273	TiC_0.3_N_0.7_
**P-1500A**	42.25	0.214	0.4273	TiC_0.4_N_0.6_
**P-1600NH**	42.6	0.212	0.4245	TiN
**P-1600N**	42.2	0.214	0.4283	TiC_0.4_N_0.6_
**P-1600A**	42.1	0.215	0.4293	TiC_0.6_N_0.4_
**P-1800NH**	42.6	0.212	0.4245	TiN
**P-1800N**	42.15	0.214	0.4288	TiC_0.5_N_0.5_
**P-1800A**	41.9	0.216	0.4312	TiC_0.8_N_0.2_

## Supplementary Materials

The following are available online, Figure S1: FTIR spectra of PVMSZ, PVMSZ_T and PVMSZTi*2.5* samples, Figure S2: liquid-state ^13^C-NMR spectrum of TDMAT, Figure S3: experimental ^13^C CP MAS NMR recorded for the PVMSZTi*2.5* sample from −20 to 160 ppm, Figure S4: TG curves recorded during decomposition of PVMSZ in flowing ammonia, nitrogen and argon, Figure S5: experimental ^29^Si CP MAS NMR for the **P-1400NH**, **P-1400N, P-1400A** and **P-1800A** samples at 7 T, Figure S6: identification of the α and β-Si_3_N_4_ phases in the XRD pattern of **P-1500N**, Figure S7: HRTEM micrographs of **P-1500NH** (a), **P-1500N** (b) and **P-1500A** (c) samples with FFT images obtained from HRTEM images of **P-1500N** (b) and **P-1500A** (c) samples as insets, Figure S8: EDS mapping of the **P-1600A** sample, Figure S9: EDS mapping of the **P-1800A** sample.
