# Supplementary material for: In-Situ Synthesis and Characterization of Nanocomposites in the Si-Ti-N and Si-Ti-C Systems
Source: Molecules. 2020 Nov 10;25(22):5236. doi: 10.3390/molecules25225236 (PMC7696609; doi:10.3390/molecules25225236)
Supplement: Supplementary file 1 [file molecules-25-05236-s001.pdf]

ESI

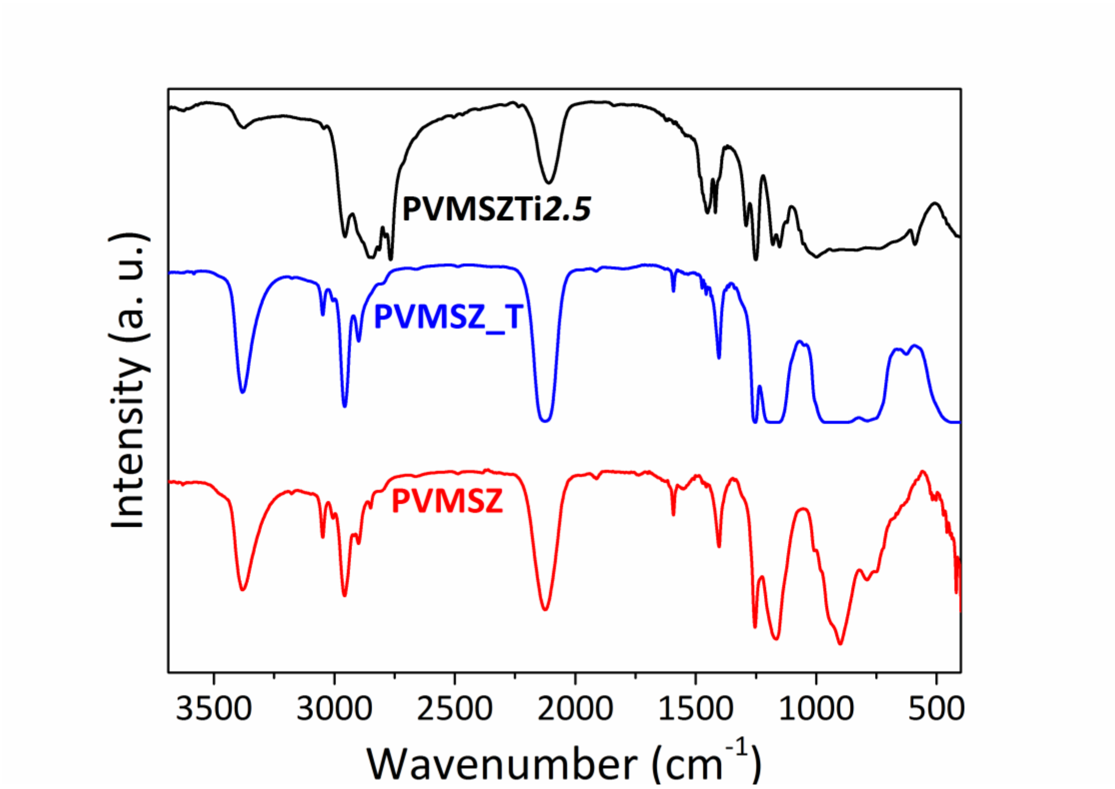

Figure S1: FTIR spectra of PVMSZ, PVMSZ_T and **PVMSZTi2.*5*** samples.

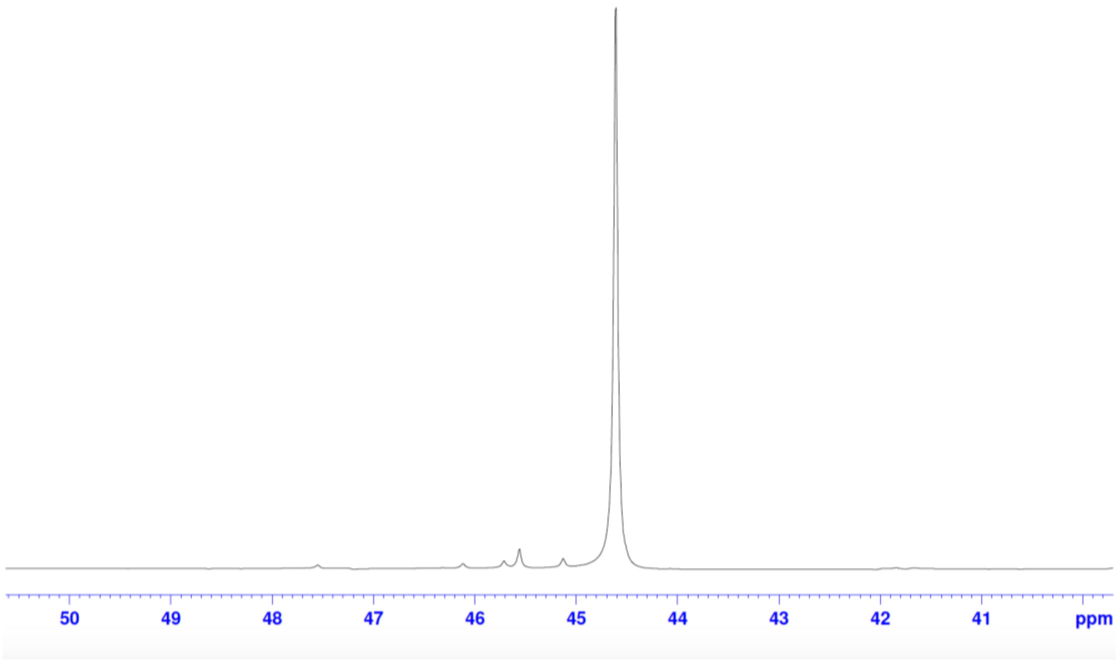

Figure S2: liquid-state ^{13}C NMR spectrum of TDMAT.

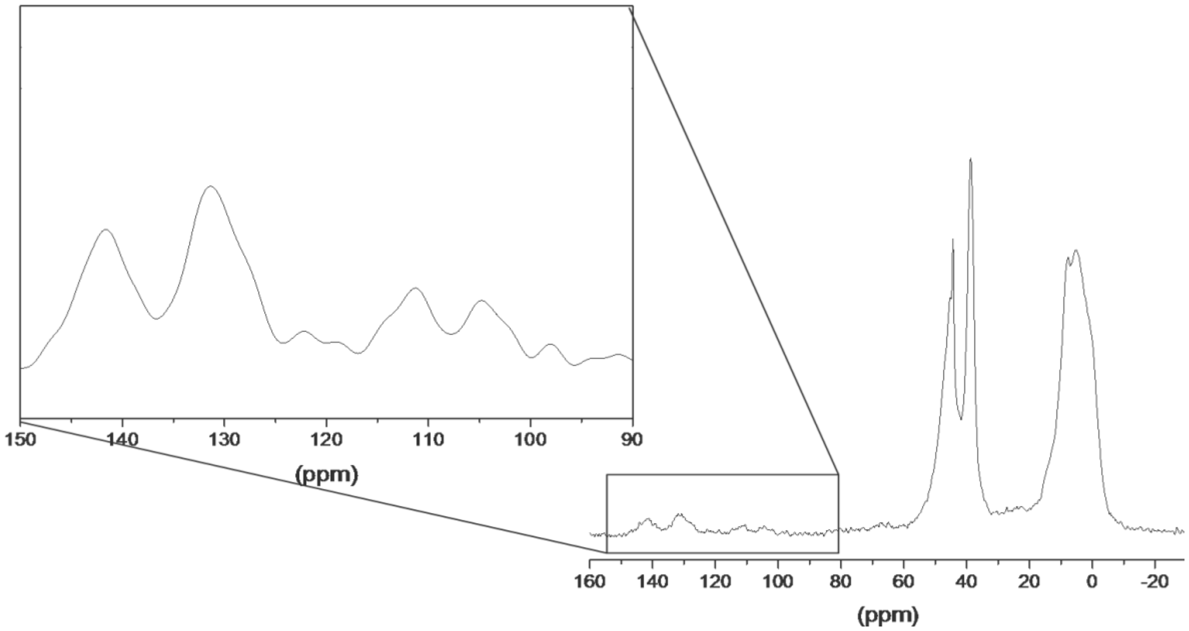

Figure S3: experimental ^{13}C CP MAS NMR recorded for the **PVMSZTi2.5** sample from -20 to 160 ppm.

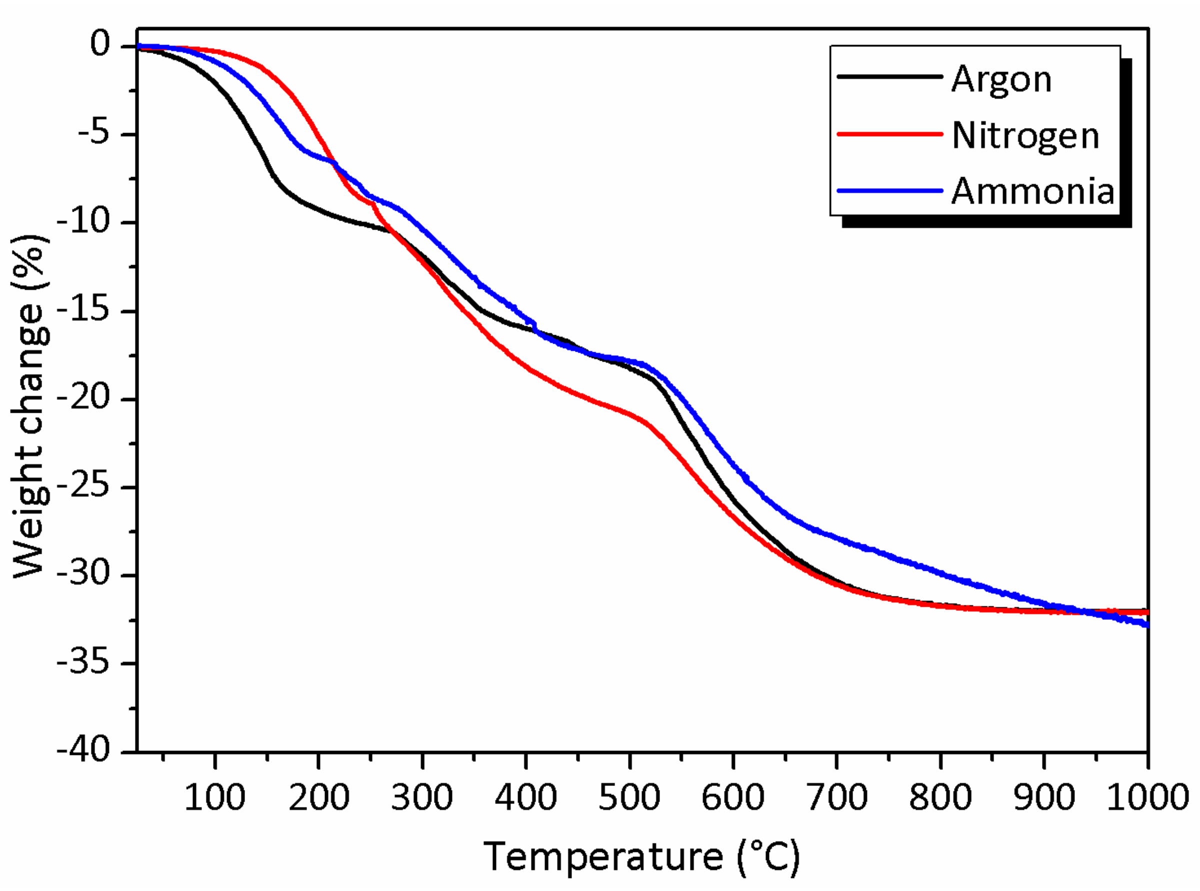

Figure S4: TG curves recorded during decomposition of PVMSZ in flowing ammonia, nitrogen and argon.

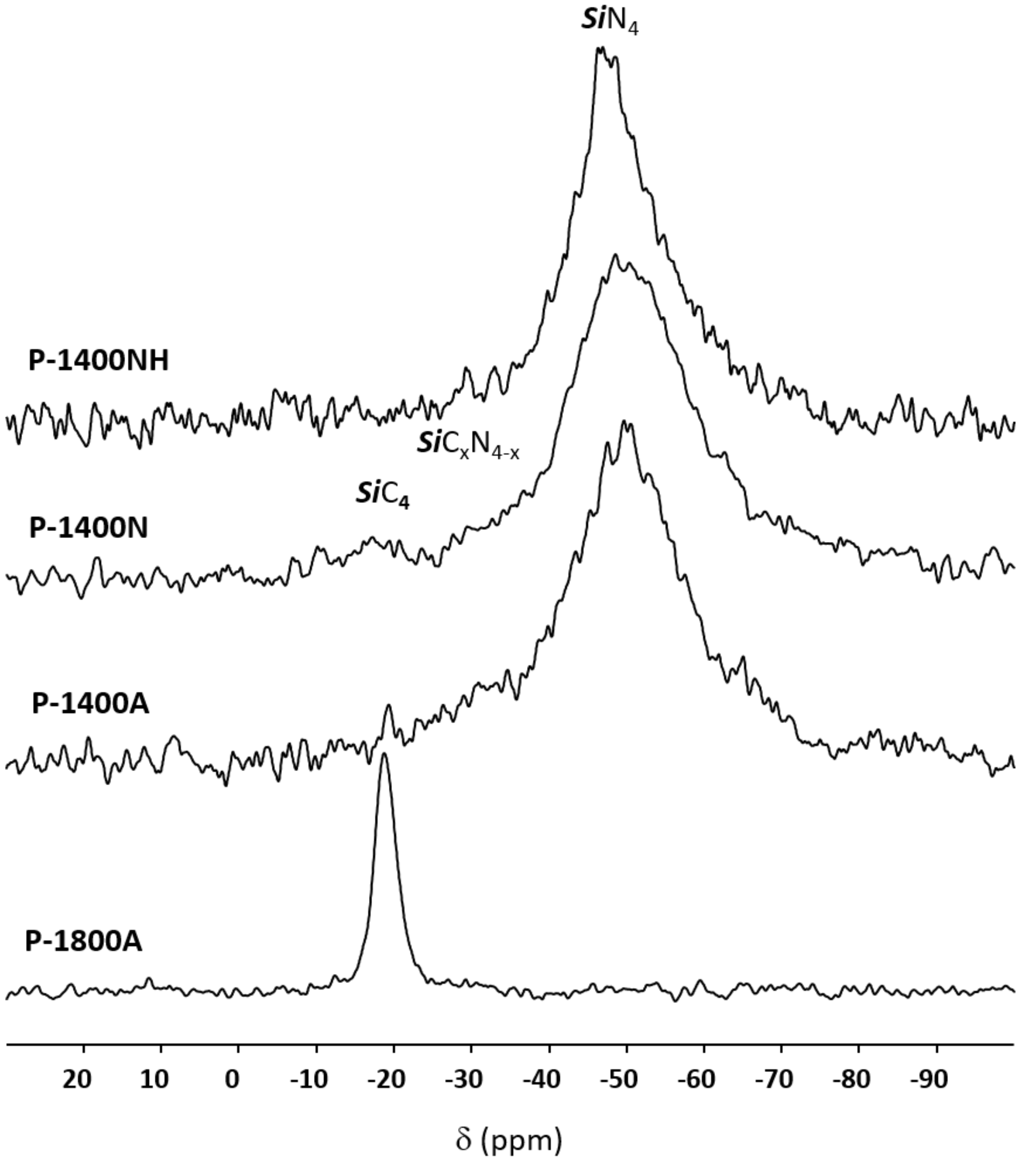

Figure S5: experimental ^{29}Si CP MAS NMR for the **P-1400NH**, **P-1400N**, **P-1400A** and **P-1800A** samples at 7 T.

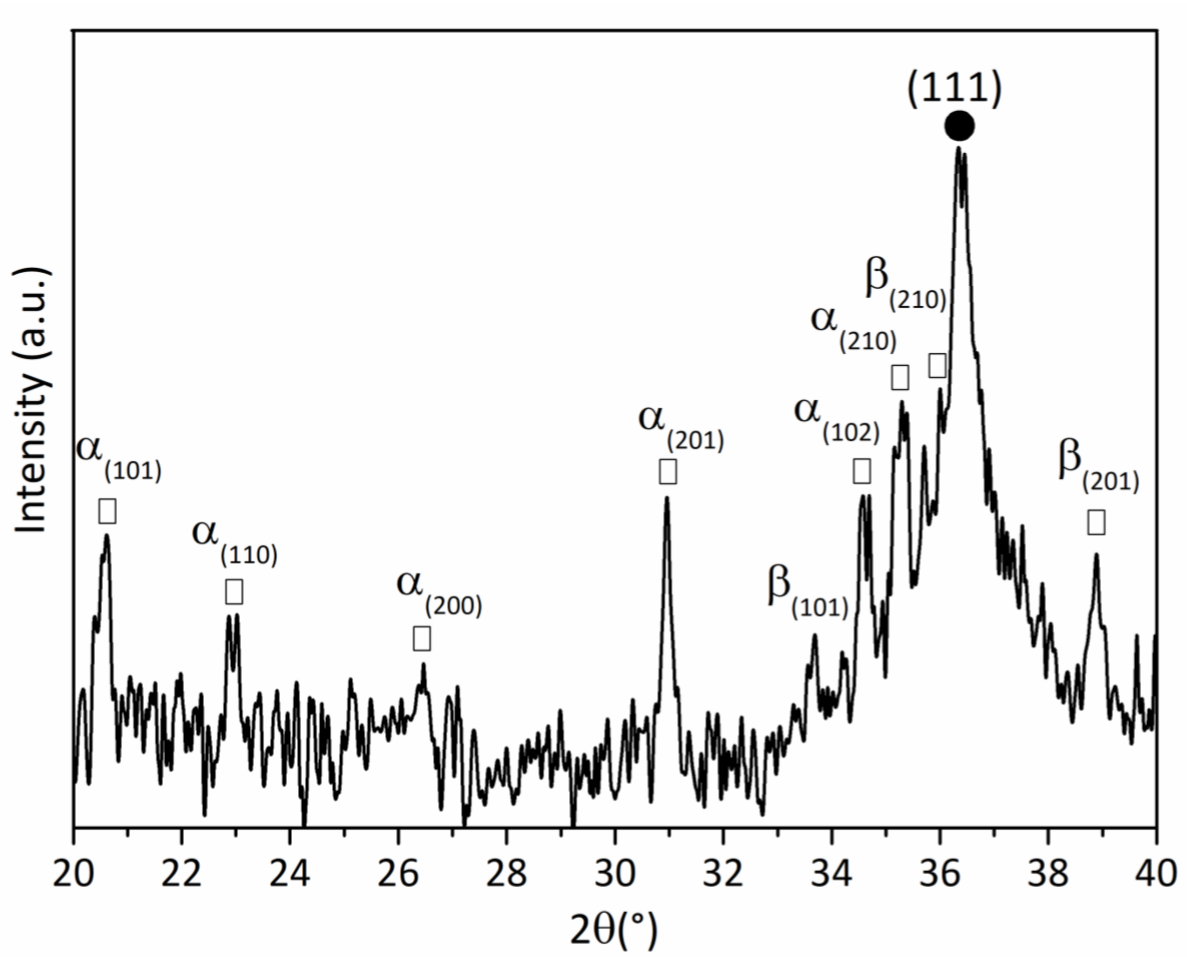

Figure S6: identification of the α and β-Si_3N_4 phases in the XRD pattern of **P-1500N**.

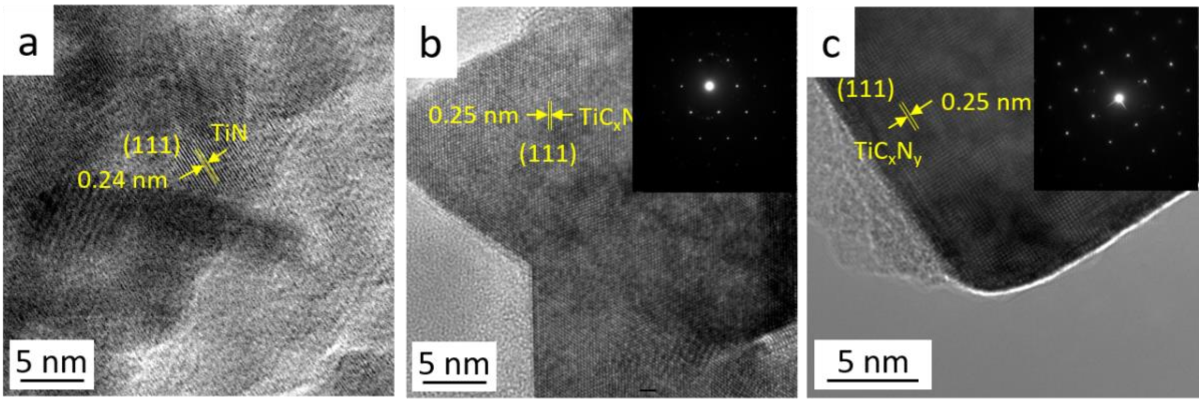

Figure S7: HRTEM micrographs of **P-1500NH** (a), **P-1500N** (b) and **P-1500A** (c) samples with FFT images obtained from HRTEM images of **P-1500N** (b) and **P-1500A** (c) samples as insets.

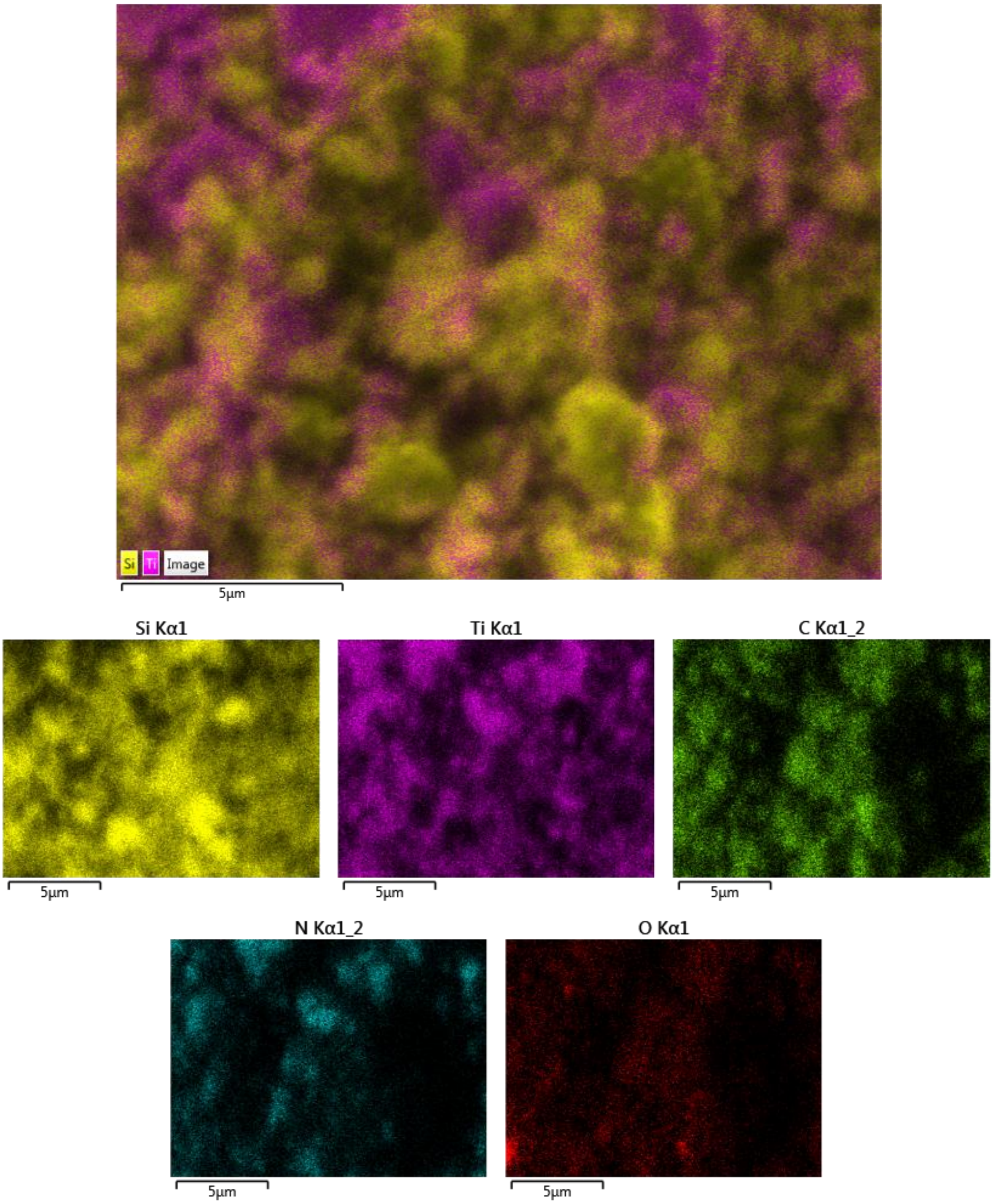

Figure S8: EDS mapping of the **P-1600A** sample.

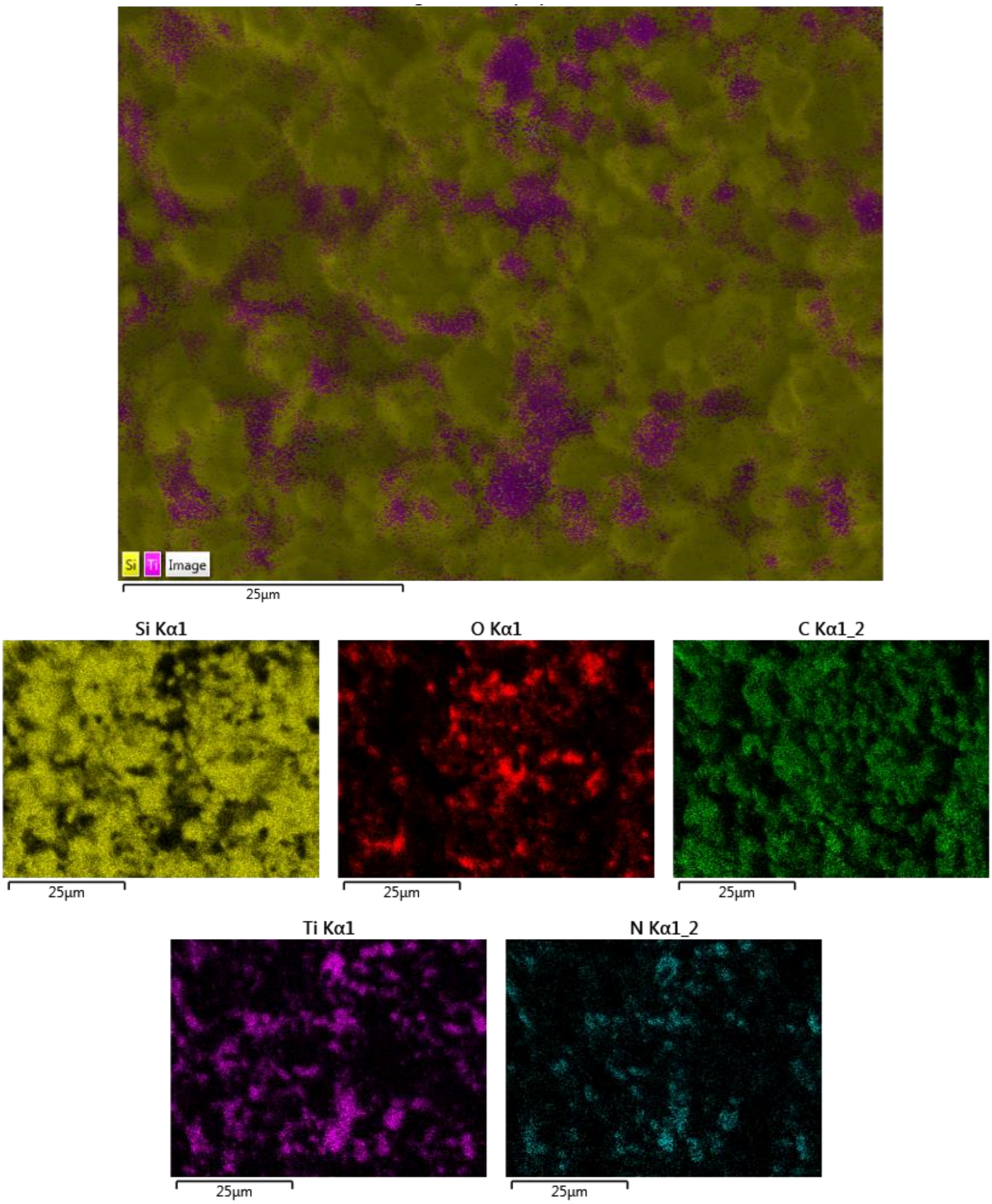

Figure S9: EDS mapping of the **P-1800A** sample.
